# Differential labelling of human sub-cellular compartments with fluorescent dye esters and expansion microscopy†

**DOI:** 10.1039/d3nr01129a

**Published:** 2023-11-09

**Authors:** Thomas M. D. Sheard, Tayla B. Shakespeare, Rajpinder S. Seehra, Michael E. Spencer, Kin M. Suen, Izzy Jayasinghe

**Affiliations:** a School of Biosciences, Faculty of Science, University of Sheffield Sheffield S10 2TN UK t.sheard@sheffield.ac.uk; b School of Molecular and Cellular Biology, University of Leeds LS2 9JT UK; c EMBL Australia Node in Single Molecule Science, School of Biomedical Sciences, University of New South Wales Sydney Australia i.jayasinghe@sheffield.ac.uk

## Abstract

Amine-reactive esters of aromatic fluorescent dyes are emerging as imaging probes for nondescript staining of cellular and tissue architectures. We characterised the staining patterns of 14 fluorescent dye ester species with varying physical and spectral properties in the broadly studied human HeLa cell line. When combined with the super-resolution technique expansion microscopy (ExM) involving swellable acrylamide hydrogels, fluorescent esters reveal nanoscale features including cytoplasmic membrane-bound compartments and nucleolar densities. We observe differential labelling patterns linked to the biochemical properties of the conjugated dye. Alterations in staining density and compartment specificity were seen depending on the timepoint of application in the ExM protocol. Additional complexity in labelling patterns was detected arising from inter-ester interactions. Our findings raise a number of considerations for the use of fluorescent esters. We demonstrate esters as a useful addition to the repertoire of stains of the cellular proteome, whether applied either on their own to visualise overall cellular morphology, or as counterstains providing ultrastructural context alongside specific target markers like antibodies.

## Abstract 2

14 fluorescent NHS esters of different hydrophobicity and charges were shown to differentially label a range of subcellular compartments in HeLa cells using the super-resolution imaging technique expansion microscopy.

## Graphical abstract

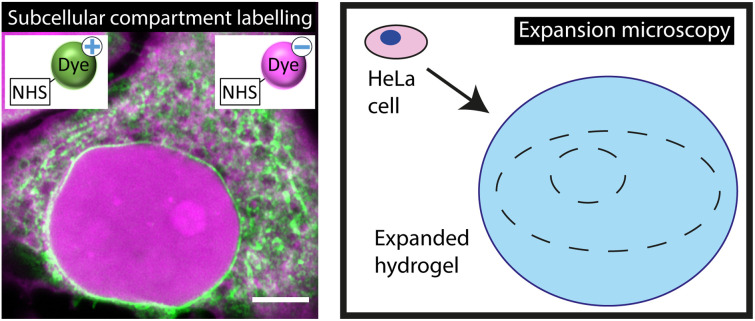

## Introduction

Modern microscopy techniques offer a view into the finest of length-scales in which the fundamental mechanisms of life operate. Variants of electron microscopy (EM) and super-resolution optical microscopies have been the primary tools for visualising the nanometre-scale features of biomolecular assemblies, cytoskeletons, membrane-bound and membraneless compartments in cells. Due to the lack of intrinsic contrast in most biological samples, staining techniques are required to delineate the structures within. Staining strategies are either passive (for instance heavy metal stains for EM) or targeted (such as immuno-labelling and click chemistry strategies). Super-resolution techniques particularly rely on the contrast and specificity offered by targeted fluorescent probes.

A recent optical technique called expansion microscopy (ExM) achieves improved resolution by physically expanding an imprint of the sample inside a swellable acrylamide hydrogel,^1^ enabling super resolution (10–50 nm) imaging with diffraction-limited microscopes. This tissue clearing approach involves proteolytic digestion of the sample (necessary to allow isotropic expansion) which effectively ‘de-crowds’ molecular structures, significantly reducing levels of background signal and thus enhancing targets of interest. ExM, like other super-resolution microscopy approaches, has enabled insights into the nanoscale distribution of molecular targets, but until recently has proven less useful for localizing nanoscale structures in the context of the broader cellular architecture.

A novel labelling strategy to visualise cellular compartments entails the use of amine reactive *N*-hydroxysuccinimide (NHS) esters, used commonly for fluorescently tagging purified proteins such as antibodies. When applied to biological samples (cells, tissues, or whole organisms), these fluorescent esters produce a nondescript staining of the entire proteome, allowing for the interpretation of compartments such as organelles, or the ability to differentiate between cell and tissue types. NHS esters were recently introduced in pan-ExM,^2^ wherein 16-fold expansion allowed fine cellular ultrastructure such as mitochondrial cristae, golgi cisternae, and nucleolar sub-compartments to be seen. Several groups have used NHS esters with ExM to understand cellular structures,^3–7^ with many others implementing esters alongside direct protein labels in order to understand the arrangement of specific molecular targets.^8–15^ More recently, one nanometer ExM used NHS esters to enable unprecedented visualisations of the conformations of individual proteins.^16^ The physical properties of the conjugated dye have been hypothesised as a major determinant of the labelling patterns, dictating which cellular structures are illuminated.^7,12^ While the rapid uptake of fluorescent esters in the ExM community shows the promise of these labels, there remains to be an evaluation of a wider range of fluorescent esters in a broadly used cell line. Furthermore, it is unclear which factors impact labelling patterns, limiting the reproducibility, validation, and broader uptake of these probes within the bioimaging community.

In this report, we characterise the labelling patterns of a variety of fluorescent esters (possessing wide-ranging physical and spectral properties) in HeLa cells. We observed differential compartment labelling between different ester dye families. Fluorescent esters provided useful contextual information as a counterstain alongside antibody staining for specific molecular targets at organelles including mitochondria, golgi and endoplasmic reticulum (ER). We report complexities of the staining patterns relating to aspects of the protocol including timing of addition, as well as multi-ester interactions, highlighting important considerations for their use as stains when imaging human cells.

## Results

### Catalogues of fluorescent esters from different dye families

**Fig. 1:**
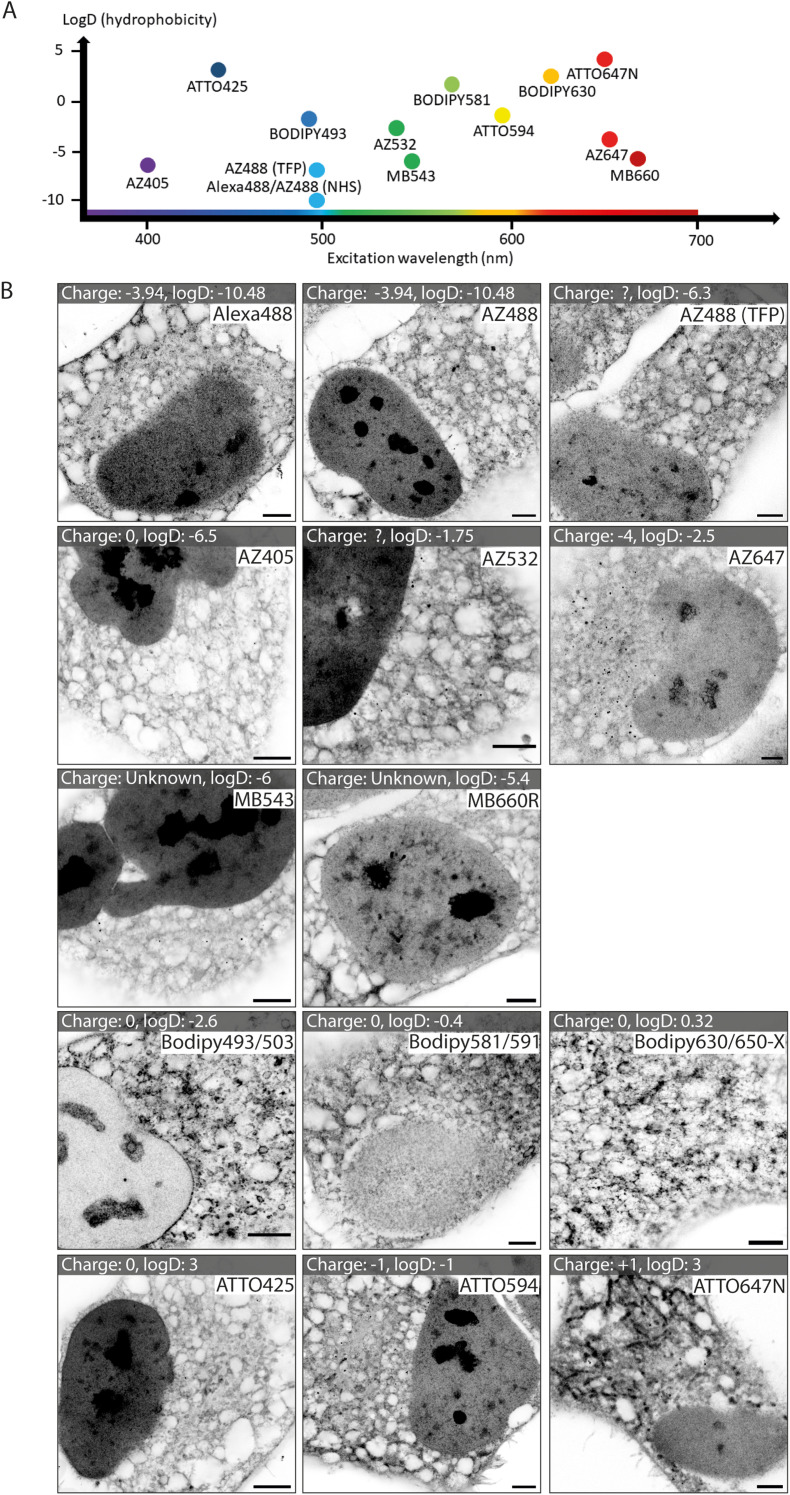
Imaging HeLa cell staining patterns of fourteen dye esters with 4× expansion microscopy. (A) Chart displaying the ester dyes, distributed based on their excitation wavelength and hydrophobicity (determined from log *D* values). (B) A gallery of expanded images of each of the esters in HeLa cells, whereby each ester is added pre-gelation. Between different dye families there are substantial differences, and within a single dye family there are similarities in patterns. Scale bars (expansion factor rescaled): 2.5 μm.

We tested a selection of 14 fluorescent ester species belonging to different dye families (Alexa Fluor, AZ, MB, BODIPY and ATTO), each with distinct charges, hydrophobic properties, and spectral emission ranges (Fig. 1A). Hydrophobicity is predicted from the distribution coefficient log *D* value (listed in Table 1) which is calculated using the structural formula of each ester. Dye esters with more negative log *D* values are more hydrophilic; dye esters with more positive log *D* values are hydrophobic. A catalogue of Airyscan images shows the labelling patterns for each ester in fixed HeLa cells after 4× expansion (Fig. 1B). The 4× enhanced ExM (EExM) approach combines the 4-fold resolution improvement from ExM with the resolution-doubling of Airyscan microscopy to achieve an effective resolution of ∼40 nm.^17,18^

**Table 1:** Ester properties

Dye	Exc. max (nm)	Em. max (nm)	Hydrophobicity (log *D*)	Overall charge	Source
NHS Alexa488	494	517	(−10.48)^a^	(−3.94)^a^	Thermo Fisher Scientific
NHS AZ488	490	525	(−9.4)^e^	(−3.94)^a^	Fluoroprobes
TFP AZ488	495	515	(−6.3)^e^	Unknown	Fluoroprobes
NHS AZ405	401	421	(−6.5)^e^	Unknown	Fluoroprobes
NHS AZ532	532	554	(−3.26),^b^ (−1.75)^e^	Unknown	Fluoroprobes
NHS AZ647	650	665	(−6.72),^b^ (−2.5)^e^	−(4)^c^	Fluoroprobes
NHS BODIPY493/503	493	503	(−2.6)^e^	0	Thermo Fisher Scientific
NHS BODIPY581/591	581	591	(−0.4)^e^	0	Thermo Fisher Scientific
NHS BODIPY630/650-X	625	640	(0.32)^e^	0	Thermo Fisher Scientific
NHS ATTO425	439	485	(3)^e^	(0)^d^	Sigma-Aldrich
NHS ATTO594	597	625	(−1)^e^	(−1)^d^	Sigma-Aldrich
NHS ATTO647N	645	669	(1.96),^a^ (3)^e^	(1),^d^ (0.61)^a^	Sigma-Aldrich
NHS MB543	543	566	(−6)^e^	1 more negative sulfo group *vs.* equivalent Alexa dye	Fluoroprobes
NHS MB660R	673	694	(−5.4)^e^	1 more negative sulfo group *vs.* equivalent Alexa dye	Fluoroprobes

a Zanetti-Domingues *et al.*, (2013).^29^

b Hughes *et al.*, (2014).^30^

c Zhang *et al.*, (2017).^23^

d https://www.spectra.arizona.edu/supplemental/ATTO_Dye_Properties_01.pdf, accessed 15/02/2023.

e Calculated using Chemaxon log *D* predictor.

Alexa dyes (and their derivatives) possess a negative charge due to sulfonation, and are generally hydrophilic.^19^ Our analysis included three direct derivatives of fluorescein: NHS Alexa488 (the most hydrophilic of the dyes tested; see Table 1), NHS AZ488, and a tetrafluorophenyl (TFP) ester of AZ488. The staining morphologies of each of these fluorescent ester species (Fig. 1B, first row) featured a broad reticular morphology throughout the cytoplasm, as anticipated with the cell's ER network. Also observed, was a high intensity of staining of the nucleoplasm by comparison to the cytoplasm. Staining within each nucleus include dense staining of several distinct nucleolar regions. These staining patterns were broadly consistent with images acquired at the pre-expansion stages (ESI Fig. 1,† first row).

Other AZ dyes included moderately hydrophilic NHS AZ405, NHS AZ532 and NHS AZ647, which reported distinctly different weightings of labelling density between the nuclear and cytoplasmic reticular elements (Fig. 1B, second row). MB dyes are another Alexa derivative type featuring an additional negatively charged sulfo-group (improving water solubility and minimising self-quenching) and are predicted to be similarly hydrophilic. The labelling patterns of NHS MB543 and NHS MB660R in the cytoplasm were also web-like, albeit possessing different densities, and more concentrated to a region in proximity to the nucleus in unexpanded cells (ESI Fig. 1,† third row). NHS MB543 often strongly stained the nucleoplasm compared to the cytoplasm.

The BODIPY family of dyes is strongly hydrophobic with zero net charge, and has been effectively used as imaging probes to observe lipid-dense structures.^20^ ExM images of HeLa cells stained with these fluorescent esters (NHS BODIPY493/503, NHS BODIPY581/591 and NHS BODIPY 630/650-X) highlighted a strikingly different component of labelling to that seen with the Alexa, AZ, and MB ester species (Fig. 1B, fourth row). Staining of the nucleoplasmic regions was either absent or modest, whilst the reticular morphology in the cytoplasm was more variable in intensity, and punctuated by compact, high-intensity structures that resembled lipid droplets, endosomes or other small organelles.

Finally, the NHS ATTO425, NHS ATTO594 and NHS ATTO647N dye esters typically possess more positive log *D* values (Table 1) than Alexa dyes, indicating an overall more hydrophobic tendency. However, they vary in net charge from 0, −1, and +1 respectively. All of the ATTO esters labelled dense structures within the nucleus, while in the cytoplasm the patterns were more akin to those from the Alexa family than the BODIPY family (Fig. 1B, bottom row). However, specific features such as elongated bodies of intense staining that resembled mitochondria were prominently observed with NHS ATTO647N (replicating the observation of ref. 21), and to a lesser extent with NHS ATTO594.

### Differential compartment labelling with fluorescent esters

**Fig. 2:**
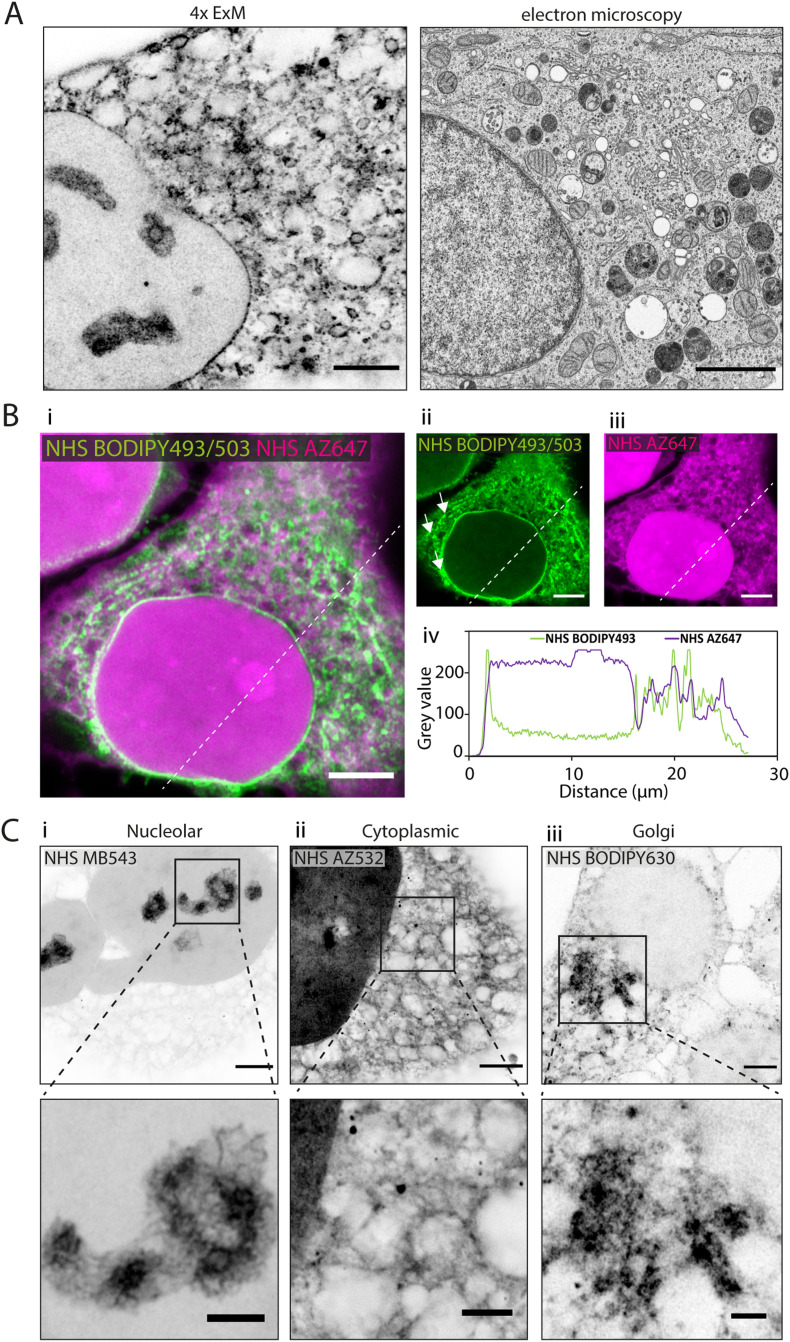
Differential subcellular labelling patterns of directly applied fluorescent esters observed in expansion microscopy. (A) Intracellular compartments can be observed in a HeLa cell labelled with NHS BODIPY493/503 (applied pre-gelation) and imaged with 4× EExM (left). The level of spatial detail is comparable to that in a 2D transmission electron micrograph (right). Image adapted from Hennies *et al.* with permission (Hennies *et al.*, 2020).^22^ (B) Pre-expansion images show how ester labelling patterns differ depending on the properties of the conjugated dye. (i–iii) The hydrophilic NHS AZ647 (magenta) labels the nucleus more strongly than the hydrophobic NHS BODIPY493/503 (green) as indicated by the increased intensity shown in the line profile chart (iv). (C) 4× EExM images demonstrate examples of the sub-cellular compartments that can be preferentially labelled by esters, including (i) nucleoli (NHS MB543 applied pre-gelation), (ii) the complex web-like appearance of membranes and organelles in the cytoplasm (NHS AZ532 applied pre-gelation), and (iii) dense regions adjacent to the nucleus belonging to the golgi apparatus (NHS BODIPY630 applied post-digestion). Scale bars (expansion factor rescaled): (A) (left) 2.5 μm, (right) 2 μm; (B) 10 μm, (C) (upper) 2.5 μm, (lower) 1 μm.

In 4× EExM images, the hydrophobic NHS BODIPY493/503, with a net charge of 0, yielded a varied impression of a number of the major cellular components of the HeLa cells (Fig. 2A, left) including a well-resolved nuclear envelope, reticular organelles such as the ER, and micro-organelles such as endosomes, lipid droplets, and mitochondria. These features compare closely with an exemplar thin-section electron micrograph by Hennies *et al.*^22^ shown to scale (Fig. 2A, right).

When unexpanded HeLa cells were dual-stained with two spectrally-distinct esters possessing opposing biochemical properties, differential views of cellular compartments were obtained (Fig. 2B-i). The hydrophobic NHS BODIPY493/503 (green, Fig. 2B-ii) and hydrophilic NHS AZ647 (magenta, Fig. 2B-iii) co-staining reported weakly correlated labelling of non-nuclear compartments (Fig. 2B-iv). The most striking difference between the patterns was the intense intra-nuclear labelling with NHS AZ647, in contrast to the lipophilic NHS BODIPY493/503 which labelled the nuclear envelope. The labelling patterns found in the cytoplasm were extensive in both channels, with considerable overlapping and non-overlapping elements. For example, NHS BODIPY493/503 labelled elongated structures adjacent to the nucleus (arrowheads in Fig. 2B-ii) which were not observed as clearly with NHS AZ647.

Differential staining morphologies were further examined with 4× EExM of a chosen subset of NHS ester dyes, showing highly preferential staining of different cellular regions. These included nucleolar structures and the chromatin-associated proteome (Fig. 2C-i, NHS MB543 pre-gelation), complex cytoplasmic meshes of various organelles (Fig. 2C-ii, NHS AZ532 pre-gelation), and the Golgi apparatus located adjacent to the nucleus (Fig. 2C-iii, NHS BODIPY630 post-digestion).

### Fluorescent esters as counterstains alongside antibody-labelled structures

**Fig. 3:**
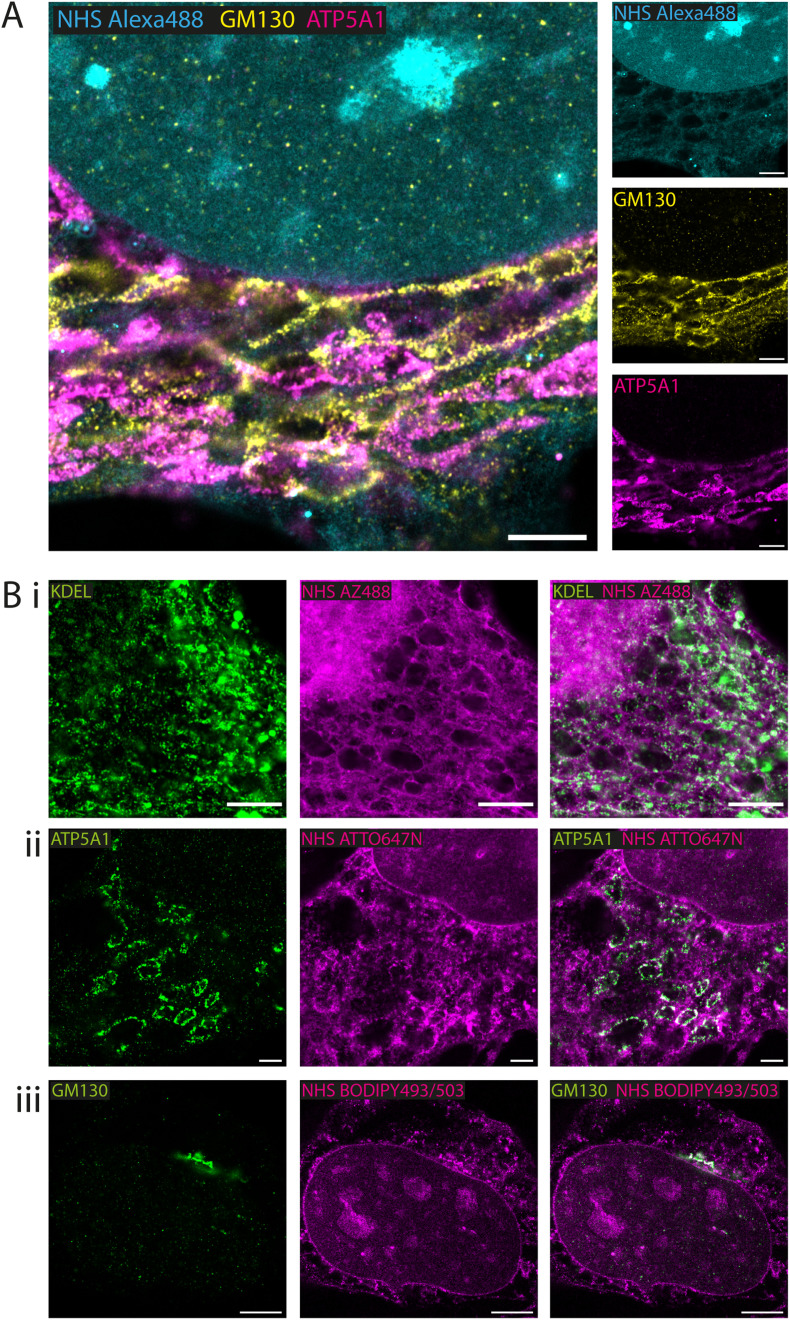
Fluorescent esters as counterstains alongside antibody benchmarks. Combining NHS esters with immunofluorescence provided with spatial context for the localisation of the antigens in HeLa cells. (A) 4× EExM images of antibody labels for the *cis*-golgi marker GM130 (yellow) and the mitochondrial ATP5A1 ATP synthase (magenta) alongside NHS Alexa488 (cyan) applied pre-gelation. (B) 4× EExM images shows examples of antibody labelling for targets at specific compartments alongside ester counterstains (all applied pre-gelation), including (i) the KDEL endoplasmic reticulum retention sequence, alongside NHS AZ488 revealing aspects of the cytoplasmic reticular network and organelles, (ii) the mitochondria ATP synthase subunit ATP5A1 alongside NHS ATTO647N, and (iii) the *cis*-golgi matrix protein GM130 alongside NHS BODIPY493/503. Scale bars (expansion factor rescaled): (A) 3.75 μm; (Bi) 2.5 μm, (Bii) 2.5 μm, (Biii) 5 μm.

Whole proteome staining with NHS esters is rapidly emerging as an insightful counterstain to the localisation of targeted markers such as antibodies. Fig. 3A features exemplar 4× EExM images from a triple stain of the Golgi apparatus (*via* anti-GM130; yellow) and mitochondria (*via* anti-ATP synthase ATP5A1; magenta) against a counterstain of NHS Alexa488 (cyan). The Golgi network and the mitochondrial staining were seen to occupy largely non-overlapping cytoplasmic regions. The reticular staining of NHS Alexa488 indiscriminately occupied regions that were stained either for Golgi or mitochondrial targets, indicating that the composite nature of the cytoplasmic staining by esters relates to multiple different organelles and ultrastructural elements.

A series of 4× EExM images illustrates compartment-specific antibody stains visualised against NHS esters selected based on the greatest weighting of staining to the compartment of interest. The ER protein retention tag, targeted using anti-KDEL antibodies (green), labelled elements of the ER network more distal from the nucleus as visualised alongside NHS AZ488 (Fig. 3B-i). The outlines of mitochondria were labelled with anti-ATP5A1 antibodies (Fig. 3B-ii), visualised in the context of loops of reticular components labelled with NHS ATTO647N. Finally, the *cis*-golgi was targeted using anti-GM130 antibodies, and appeared to be preferentially labelled by the hydrophobic NHS BODIPY493/503, which was also closely positioned adjacent to the nuclear membrane (Fig. 3B-iii).

### Considerations affecting fluorescent ester staining morphologies

#### Application at different time-points of the ExM protocol

The timing of application of fluorescent NHS esters has been hypothesised to impact their compartment labelling preferences,^7^ relating to the presence of the hydrogel and the decrowding effect from proteolytic digestion and expansion. To investigate this, we applied NHS ATTO647N within separate samples at three time-points: pre-gelation, inter-digestion (4 hours of proteinase digestion, followed by NHS ester application, and subsequent digestion of 4 hours), and post-digestion (ESI Fig. 2A†). When applied pre-gelation NHS ATTO647N stained elongated compartments surrounding the nuclei that bore resemblance to mitochondria, with a moderate density of staining also present in the nucleoplasm (ESI Fig. 2B†). In contrast, when applied inter-digestion the labelling was completely excluded from the nucleoplasm, and the cytoplasmic labelling appeared at discrete protein densities (resembling rough ER or small vesicles). The labelling pattern observed pre-gelation was completely reversed when the same dye ester was applied post-digestion, most strongly labelling the nucleus. The cytoplasmic labelling in these samples appeared weaker in intensity and the protein densities sparser.

#### Interactions between multiple fluorescent esters

**Fig. 4:**
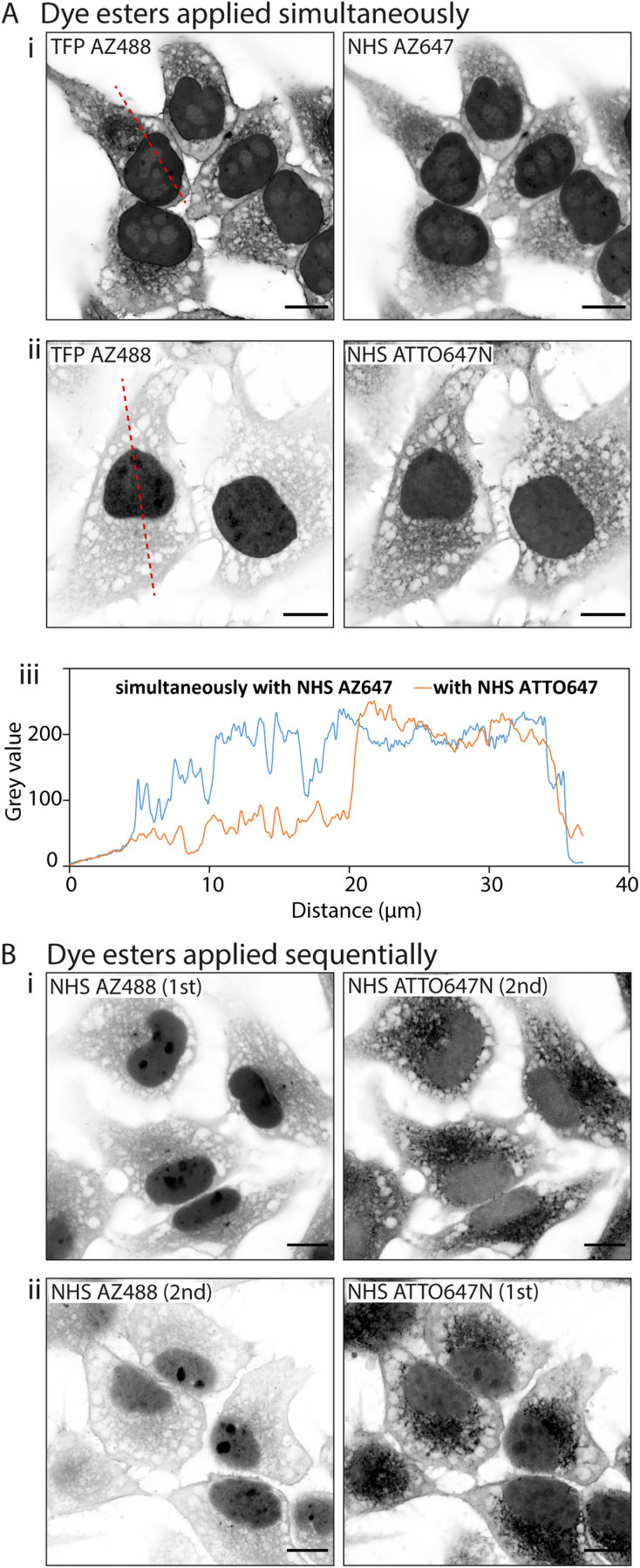
Interactions between multiple fluorescent ester species. When multiple fluorescent esters are added into a single HeLa cell sample, interactions between esters alter their labelling patterns. (A) Unexpanded images show that when esters are added simultaneously, (i) TFP AZ488 (which is hydrophilic) labels the cytoplasm and nucleus in a uniform way when paired with NHS AZ647 (also hydrophilic), (ii) but more strongly labels the nucleus when paired with NHS ATTO647N (hydrophobic). (iii) Line profile plot demonstrates the differing intensities across the cells. (B) When dye esters are added sequentially their labelling can be altered. (i) When applying NHS AZ488 first and then NHS ATTO647N second, the intensity of NHS AZ488 is much stronger than (ii) when the order of application is reversed. Scale bars: 10 μm.

We examined whether simultaneous application of two dye esters could shift their target specificity through inter-ester interactions. We dual-labelled HeLa cells with hydrophilic TFP AZ488 at the same time as one of two esters of distinctly different log *D* values. In the first instance we paired TFP AZ488 (predicted log *D* of −6.3; see Table 1) with another hydrophilic dye NHS AZ647 (predicted log *D* of −2.5). In Airyscan images we observed a strong intensity of TFP AZ488 staining of cytoplasmic and non-nuclear compartments (Fig. 4A-i). In the second experiment we applied TFP AZ488 alongside the hydrophobic NHS ATTO647N (log *D* of +3). The TFP AZ488 labelling density from the non-nuclear compartment in the latter experiment was considerably weaker in the presence of the hydrophobic dye (Fig. 4A-ii). This difference in labelling densities, particularly that of the nucleoplasm, is shown in the comparative line profile plots (Fig. 4A-iii).

To examine any role of fluorescence resonance energy transfer (FRET) between two probes in the morphologies in NHS ester multi-staining (*e.g.* NHS Alexa488 and NHS ATTO647N), we carried out bleaching of the FRET acceptor NHS ATTO647N in a small region of interest using a 633 nm laser (ESI Fig. 3A†). In post-bleach re-imaging we observed a corresponding increase in the signal intensity in the Alexa488 fluorescence by ∼85% with no apparent change to the labelling pattern itself (ESI Fig. 3B†).

To examine an impact of inter-ester interactions of hydrophilic and hydrophobic pairings, we applied the esters sequentially by labelling firstly with hydrophilic NHS AZ488, and then with the hydrophobic NHS ATTO647N (Fig. 4B-i). In a second experiment we switched the order around, first labelling with hydrophobic NHS ATTO647N followed by the hydrophilic NHS AZ488 (Fig. 4B-i). In both situations the labelling patterns of NHS ATTO647N were qualitatively unchanged. However, when applied second the NHS AZ488 stained at a lower density, most notably in the nucleus (Fig. 4B-ii).

#### Alternative cell types

Lastly, we compared the NHS ester staining morphologies in HeLa cells with that in hTERT human retinal pigment epithelial (RPE-1) cells (ESI Fig. 4†). Very strong similarities in the stain distribution and morphology between compartments was consistently observed between the cell types with NHS Alexa 488, NHS ATTO425, and NHS ATTO647N. As an exception to this trend, clear intra-nuclear and nucleolar staining was observed in RPE-1 cells with NHS BODIPY493/503 whilst these regions in HeLa cells were completely devoid of this stain.

## Discussion

### Utility of fluorescent esters as counter-stains for subcellular context

The direct application of fluorescent esters as nondescript stains has allowed optical microscopy to visualise subcellular ultrastructure,^2^ a feature previously reserved for electron microscopy. The 14 fluorescent esters used in this study considerably broadens the range of probes to be characterised. To our knowledge, this is the first comprehensive assessment of a broad range of fluorescent dye ester species as stains for ExM of a broadly used human cell line.

The mechanism(s) of differential compartment staining are yet to be fully resolved, made particularly challenging due to the complexity of labelling patterns in relation to experimental alterations (discussed below). Our observations point to multiple mechanisms at play, including the charge and hydrophobicity of the conjugated dye proposed previously,^7^ accessibility to reactive amine groups (subject to anchoring and digestion), and FRET between other fluorescent species present within the sample. Previous work has shown how dyes preferentially label certain compartments, for instance the affinity of ATTO647N for mitochondria, both as a stand-alone dye (without the reactive NHS ester) in live cell experiments,^23,24^ as well as in NHS ester form when applied with ExM.^21^ In order to better understand preferential labelling it would be desirable to obtain detailed descriptions of the cellular landscape for different biomolecules, such as that produced for lipids,^25^ characterising the hydrophobicity and charges of a range of organelles. Future insights into labelling mechanisms would be possible through *in vitro* dye ester binding studies on fractionated immuno-co-precipitation of specific cell compartments.

Ester staining patterns result from a projection of multiple, co-stained, compartment types, rather than being exclusive to specific organelles, a limitation that proteome staining strategies share with generic membrane staining protocols such as mCLING.^3^ For this reason, it is challenging to perform segmentation and subsequent quantification. However, future applications of machine learning approaches for organelle recognition, as have been effectively implemented to understand subcellular landmarks,^26^ will likely enable better understanding of how the complex labelling patterns of esters arise.

Ester stains make it possible to identify the location, geometry, and boundaries of compartments in a way that may not be fully captured by targeted labels such as antibodies. In our observation, proteome labelling with esters is most useful for enabling insights into overall cellular morphology and as a valuable counterstain alongside other molecular targets for interpreting protein localisation. The valuable contextual information provided by fluorescent esters make it possible to investigate spatial relationships of specific molecular targets (through antibody epitopes or endogenous labels) to neighbouring cellular compartments. Its utility was demonstrated in pan-ExM^2^ and ester counterstain of membraneless organelles known as P-granules.^14^

### Considerations affecting fluorescent ester labelling patterns

In addition to the properties of the fluorescent molecule directly conjugated to the ester, several factors can alter ester labelling patterns, including the timing of addition during the ExM protocol, and interactions with other fluorescent markers. Altogether, the possible variations in labelling may be viewed as additional degrees of versatility of fluorescent esters.

We observed differences in labelling patterns depending on timing of addition, as has been reported previously.^7^ The resulting morphologies likely depends on (1) varying availability of amine groups at different time-points, and (2) the fluorescence loss of dyes exposed to the gel polymerisation free radicals and digestive enzymes. Availability of amines is likely to vary in relation to the anchoring step, given that AcX reacts with amines,^27^ and also the effects of proteinase K hydrolysing peptide bonds, potentially exposing new amine groups in previously inaccessible regions, or losing protein fragments from the gel altogether.^28^ Our investigation of labelling timepoints made use of the hydrophobic NHS ATTO647N, however it is possible that more hydrophilic esters may experience different time-specific staining pattern alterations. In addition, it is well documented that certain dyes are less resistant to bleaching during the expansion process.^27^ The application of the dye esters at the post-digestion stage could therefore yield more efficient labelling. Nevertheless, these sequence-dependent differential labelling outcomes emphasise the importance of optimising experiments for each ester.

Further, we identified inter-ester interactions in co-staining experiments, wherein multiple esters added together can result in alterations to the reported morphology compared to that in single-stain experiments. While such interactions could relate to direct competition for amines or charge/hydrophobic interactions between the dyes, our data support multiple inter-ester interactions impacted by FRET between dyes and the sequence of dye application.

An additional factor that we did not investigate in this study relates to the composition of the buffers used in the ester incubation steps. It has been suggested that the pH and salt content of the buffer can influence labelling patterns^4^ which pose additional sources of staining variability.

### Up-take of fluorescent dye esters in the microscopy community

This study tested a broad palette of esters from different dye families in one of the most widely studied human cell lines, HeLa. When considering how labelling patterns could translate to alternate sample types, we showed that labelling patterns for esters observed in HeLa cells did not translate identically when implemented in RPE-1 human cell lines, however the broad staining patterns were conserved (ESI Fig. 4†).

The following esters are recommended as ideal starting candidates for adopting NHS ester counter stains. As a single stain for an overview of the sample, NHS Alexa488 is recommended due to its ability to label a range of structures, its robust brightness and fluorescence retention, as well as its easy visibility down the eyepiece. For a dual label approach, NHS Alexa488 and NHS ATTO647N make a useful pairing of hydrophilic and hydrophobic tendencies respectively to visualise different aspects of subcellular architecture. For labelling organelles with higher specificity, it is advised to survey a wider catalogue of candidate dyes, taking into account the properties of the dye and carrying out validation of staining morphologies through antibody-based dual stain experiments. In addition, benchmarking the timing and sequence of dye ester application as well as spectral and solubility interactions and buffers need to be established for the sample type of choice.

## Conclusions

We have characterised a collection of 14 fluorescent dye esters in HeLa cells, with increased resolution from ExM, and observed differential compartment labelling. We have shown the utility of esters for providing subcellular context alongside immunolabelled targets. We highlight a number of key factors which introduce complexities to ester labelling patterns, including the timing of application, as well as novel inter-ester interactions.

## Methods

### Experimental model

Key resources are listed in ESI Table 1,† specifying the source and product codes for each item.

HeLa-CCL2 cells (human cervix epitheloid carcinoma), originally sourced from the European Collection of Authenticated Cell Cultures (ECACC 93021013), were gifted to us by the Department of Infection, Immunity and Cardiovascular Disease, Medical School, University of Sheffield, Beech Hill Rd, Sheffield S10 2RX. HeLa cells were maintained in Dulbecco's Modified Eagle Medium (Thermo Fisher Scientific) (containing 10% (v/v) foetal bovine serum (Thermo Fisher Scientific) and 1% (v/v) penicillin–streptomycin (Thermo Fisher Scientific)) and were stored in an incubator with 37 °C with 5% CO_2_. Cells used in these experiments were from passage numbers between 13 and 25. Following a passage the cells were counted with a haemocytometer and made into a 75 000 cells ml^−1^ solution. 2 ml of this solution (150 000 cells total) was plated onto 22 × 22 mm glass coverslips (Menzel Gläser, thickness #1.5) in the bottom of 6-well plates, which had been coated with 0.01 mg ml^−1^ poly-d-lysine (Cultrex). Two days of culture on coverslips enabled the cells to attach and present an elongated morphology, which was preferable to observe structures in the cytoplasm.

As a second model of human cell lines, we used hTERT retinal pigment epithelial (RPE-1) cells, originally sourced from ATCC (CRL-4000) were gifted to us by Phillip Woodman, Division of Molecular & Cellular Function, Faculty of Biology Medicine and Health, University of Manchester, UK. RPE-1 cells were maintained in Dulbecco's modified Eagle's medium nutrient mixture F12 Ham (Sigma-Aldrich) with non-essential amino acids (Thermo Fisher Scientific) and 10% foetal bovine serum, and were stored in an incubator with 37 °C with 5% CO_2_. Following a passage, these cells were plated onto uncoated coverslips.

Cells on coverslips were fixed at Day 2 by immersion in 2% (v/v) paraformaldehyde (Sigma-Aldrich) made up in phosphate-buffered saline (PBS, Sigma-Aldrich) for 10 minutes at room temperature. Samples were washed three times with PBS for 10 minutes each. Fixed samples were stored until labelling experiments in storage solution (containing 0.05% (w/v) bovine serum albumin (Thermo Fisher Scientific), 0.1% (v/v) sodium azide (Sigma-Aldrich), made up in PBS) in 4 °C. Samples were used within 3 months of fixation.

### Fluorescent dye esters

Information regarding each fluorescent dye ester, including excitation and emission wavelengths, reported hydrophobicity and charge, has been summarised in Table 1. The log *D* value for each fluorescent ester was estimated as a measure of its hydrophobicity, using Chemaxon Calculator Plugin (address: https://disco.chemaxon.com/calculators/demo/plugins/logd/).

Ester stock solutions were prepared by reconstituting in DMSO (Biotium) to 10 mg ml^−1^ concentration. Stock aliquots were stored in the −20 °C freezer, within a desiccator chamber containing silica. Working solutions of dye esters were prepared by diluting the stock solution to working concentration of 10 μg ml^−1^ in ester stain solution, containing 100 mM sodium bicarbonate (Sigma-Aldrich), 1 M sodium chloride (Sigma-Aldrich), made to pH 6 in dH_2_O.

Ester labelling was performed was performed for 1 hour 30 minutes at room temperature, at various stages of the ExM protocol; either pre-gelation, inter-digestion (whereby gels were digested for 4 hours, then incubated with the dye esters, before another 4 hours of digestion), or post-digestion.

### Immunolabelling

Fixed samples were permeabilised with 0.1% (v/v) Triton X-100 (Sigma-Aldrich) in PBS for 10 minutes at room temperature and underwent a blocking step with 0.05% (v/v) Triton X-100 and 10% (v/v) normal goat serum (Thermo Fisher Scientific) in PBS, for one hour at room temperature.

For immunolabelled samples, after blocking the samples were incubated with primary antibodies overnight at 4 °C. The primary antibodies used in this study were rabbit polyclonal anti-KDEL (Thermo Fisher Scientific, code: PA1-013), rabbit monoclonal anti-GM130 (abcam, code: ab52649), and mouse monoclonal anti-ATP5A1 (Thermo Fisher Scientific, code: 43-9800). Antibodies were diluted 1 : 200 in antibody incubation solution (containing 2% (w/v) bovine serum albumin, 0.05% (v/v) Triton X-100, 2% (v/v) normal goat serum, 0.05% (v/v) sodium azide, made up in PBS).

The following day, samples were washed three times with PBS for 20 minutes each, and then incubated with secondary antibodies at room temperature for 2 hours. The secondary antibodies used were Alexa Fluor 488 (anti-mouse and anti-rabbit IgG), Alexa Fluor 594 (anti-mouse and anti-rabbit IgG), and Atto647N (anti-rabbit-IgG) diluted 1 : 200 in antibody incubation solution.

Immunolabelled samples were checked on the microscope to ensure suitable labelling quality and density prior to proceeding with the ExM protocol. Sample coverslips were attached to acrylic slides to facilitate viewing on the microscope stage.

### Expansion microscopy

Immunolabelled samples were incubated with 0.1 mg ml^−1^ acryloyl-X (Thermo Fisher Scientific) overnight at 4 °C for the anchoring step, then washed three times in PBS prior to the addition of gel solution.

4× expanding gels were prepared according to the recipe from the protein retention ExM approach.^27^ In short, monomer solution (containing 8.6% (w/v) sodium acrylate (Sigma-Aldrich), 2.5% (w/v) acrylamide (Sigma-Aldrich), 0.15% (w/v) *N*,*N*′-methylenebisacrylamide (Sigma-Aldrich), 11.7% (w/v) NaCl, PBS) was pre-made and stored in aliquots at −20 °C. Samples were incubated in monomer solution for 30 minutes at 4 °C, this was then removed before adding the polymerisation solution, made by mixing monomer solution with 0.2% (w/v) ammonium persulfate (Sigma-Aldrich), 0.2% (v/v) *N*,*N*,*N*′,*N*′-tetramethylethylenediamine (Sigma-Aldrich), and PBS. This solution was placed onto a parafilm-coated slide, between two coverslip spacers defining the dimensions of the final gel. The coverslip bearing the sample was then inverted onto the gel solution. Polymerisation was enabled at 37 °C for two hours.

Polymerised gels were removed from the coverslip chamber, cut into an asymmetric shape (to allow the correct orientation of the gel to be confirmed), and measured to obtain the pre-expansion size. Gels were transferred to a foil-coated 6-well plate for the digestion step, 8 U ml^−1^ proteinase K (New England Biolabs) diluted in digestion buffer (containing 50 mM Tris pH 8.0 (Invitrogen), 1 mM ethylenediaminetetraacetic acid (Sigma-Aldrich), 0.5% (v/v) Triton X-100, 0.8M guanidine HCl (Sigma-Aldrich) and deionised water (dH_2_O)). Digestion was performed overnight at room temperature, and then terminated by removing the digestion solution and adding dH_2_O.

Gels were transferred to foil-coated Petri dishes for the expansion step, which was achieved by five 30-minute washes in dH_2_O. Once expansion had reached a plateau, gels were measured to obtain the post-expansion size.

Prior to imaging, squares of gel were cut and loaded into imaging chambers (formed of a glass coverslip attached to an acrylic slide with a 18 × 18 mm cut-out). The imaging chamber coverslip was coated with 0.1% (v/v) poly-l-lysine (Sigma-Aldrich) for 30 minutes at room temperature, before three washes in dH_2_O.

### Image acquisition

Airyscan imaging was performed on an inverted LSM 880 Airyscan microscope (Carl Zeiss, Jena), using a 40× oil immersion 1.3 NA objective (working distance 210 μm). Airyscan imaging enables a roughly two-fold improvement in resolution over confocal imaging. Dyes were excited using the following lasers; Argon 488 nm, DPSS 561 nm and HeNe 633 nm, while emission bands were selected using the spectral detector and recorded with the 32-element GaAsP detector. Pixel sampling was 40 nm per pixel.

Additional imaging was performed on an inverted Zeiss LSM 980 Airyscan 2 microscope (Carl Zeiss, Jena), using a 40× oil immersion 1.3 NA objective, where excitation was provided by 488 nm, 561 nm and 639 nm lasers.

The FRET interaction experiment involved was performed on a HeLa sample dual labelled with NHS Alexa488 and NHS ATTO647N. A region within the NHS ATTO647N channel was bleached by illuminating the sample with the 639 nm laser at high intensity (approximately 20 times higher than usual) for two minutes.

### Image analysis

Airyscan processing, entailing pixel-reassignment and deconvolution, was performed using the Zen software. Applications of colour-tables and composite multi-channel overlays were performed in FIJI (ImageJ 1.53c). Scalebars presented on images indicate the ‘pre-expansion’ size, as they have been normalised for the expansion factor calculated from the physical gel size. The grey colour-tables of Airyscan and single-colour 4× EExM images have been inverted to bring the morphological appearances in line with that of thin-section EM. Therefore, the regions shown in darker pixels represented higher density of staining, therefore fluorescence intensity, compared to pixels that were lighter or white.

## Data availability

Further information and requests for resources should be directed to and will be fulfilled by the lead contacts, Izzy Jayasinghe Izzy.Jayasinghe@unsw.edu.au or Tom Sheard t.sheard@sheffield.ac.uk. Data will be shared by the lead contacts upon request. This study did not generate new unique reagents and does not report original code.

## Author contributions

TMDS, KMS, IJ designed research and acquired the funding required. MS, TS, RSS provided materials towards experiments. TMDS performed experiments, made primary observations, carried out data curation, and analysis. IJ provided the supervision. TMDS and IJ wrote the manuscript.

## Conflicts of interest

The authors declare no conflict of interests.

## Supplementary Material

NR-015-D3NR01129A-s001

NR-015-D3NR01129A-s002

NR-015-D3NR01129A-s003
