# Supplementary material for: Differential labelling of human sub-cellular compartments with fluorescent dye esters and expansion microscopy
Source: Nanoscale. 2023 Nov 9;15(45):18489–99. doi: 10.1039/d3nr01129a (PMC10667587; doi:10.1039/d3nr01129a)

Electronic Supplementary Material (ESI) for Nanoscale.
This journal is © The Royal Society of Chemistry 2023

NHS Alexa488

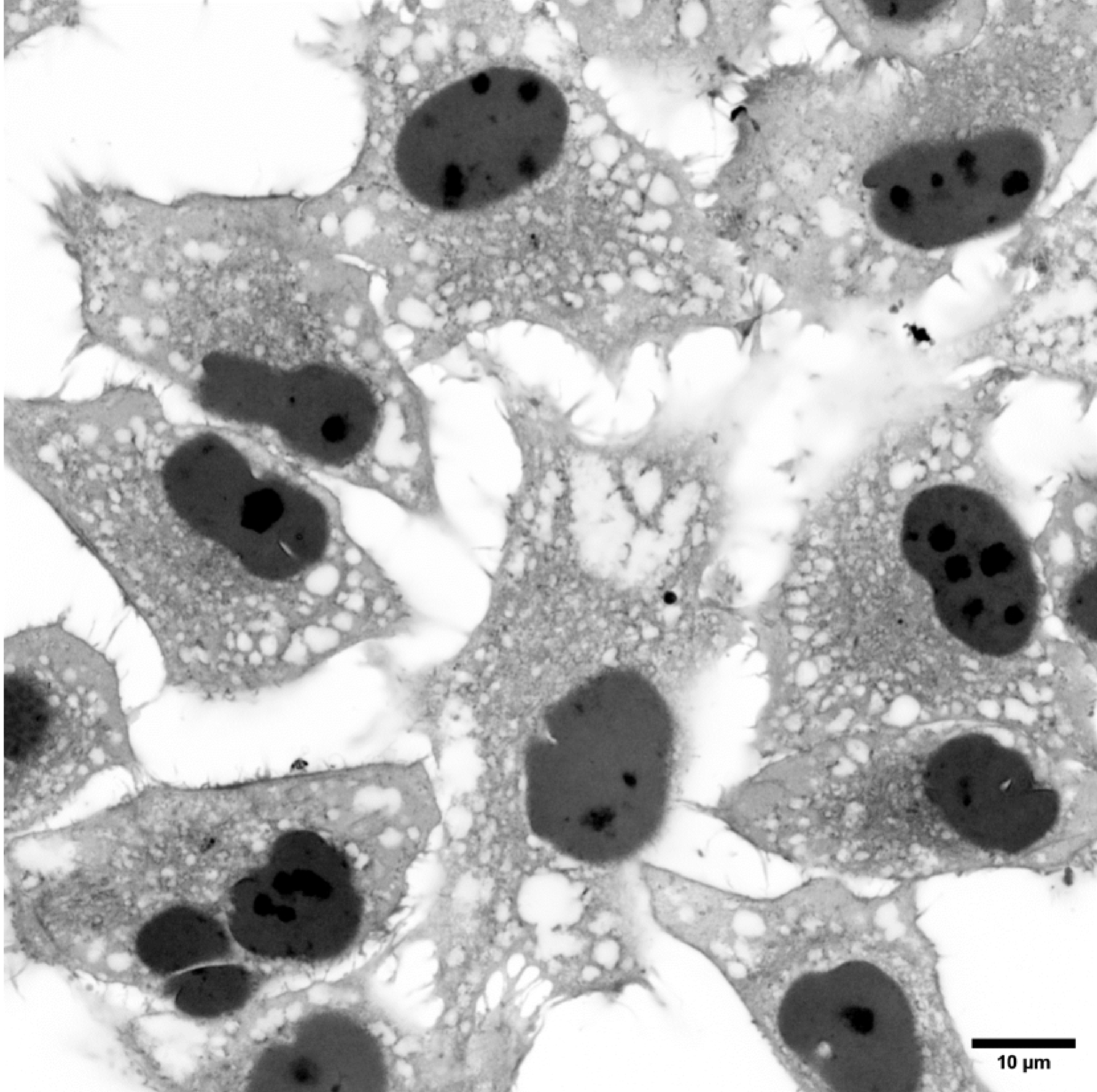

NHS AZ488

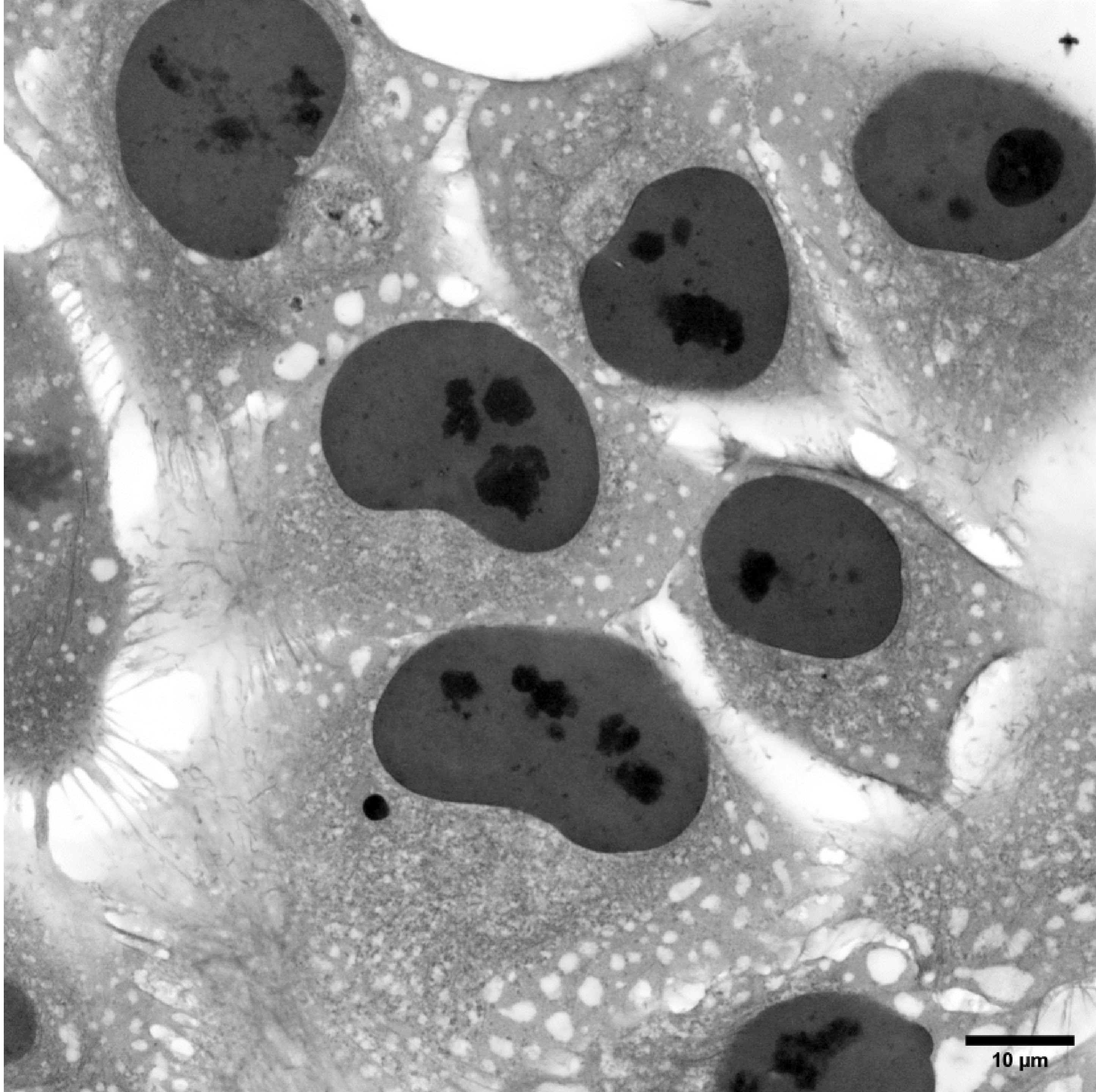

TFP AZ488

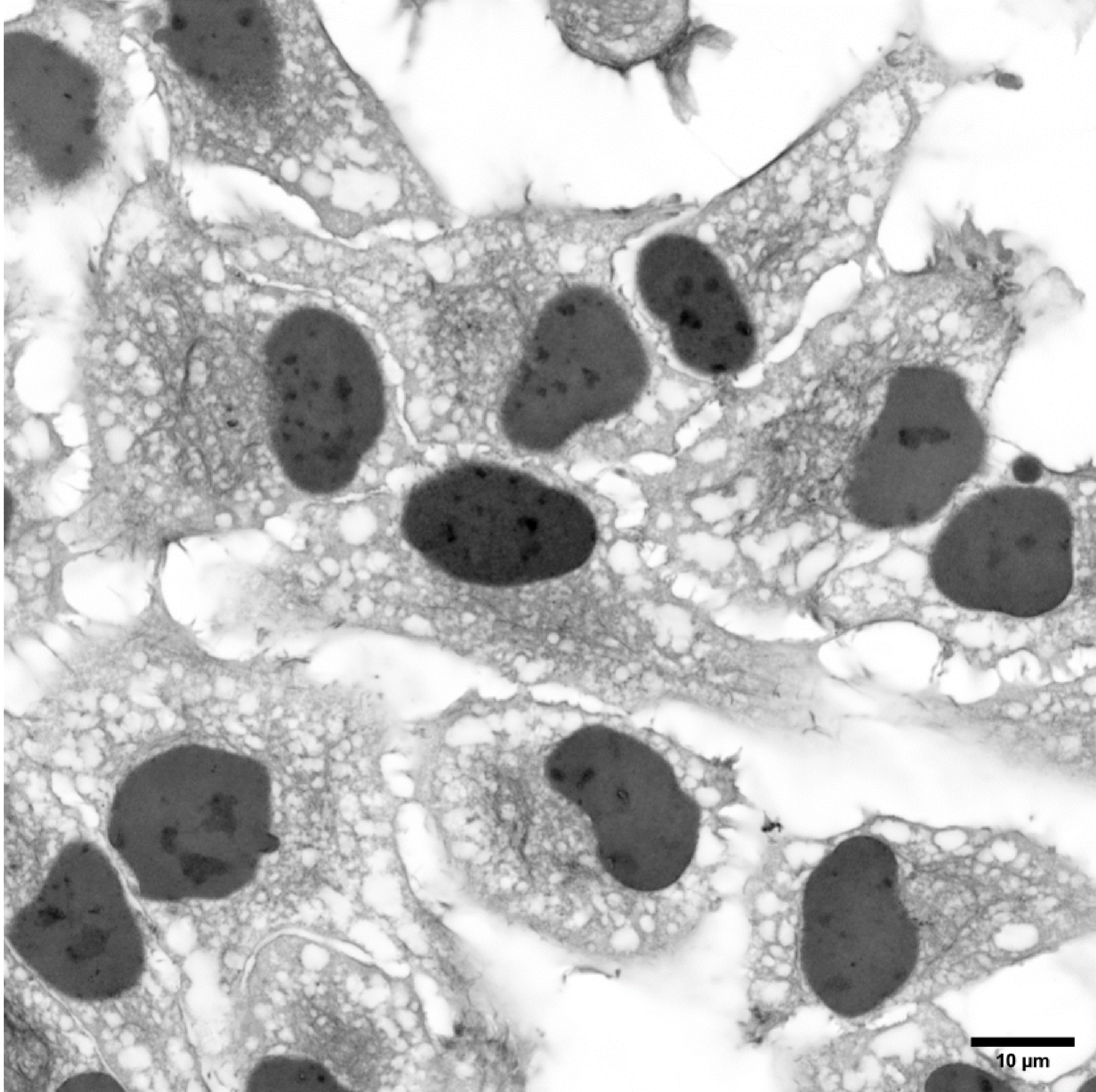

NHS AZ405

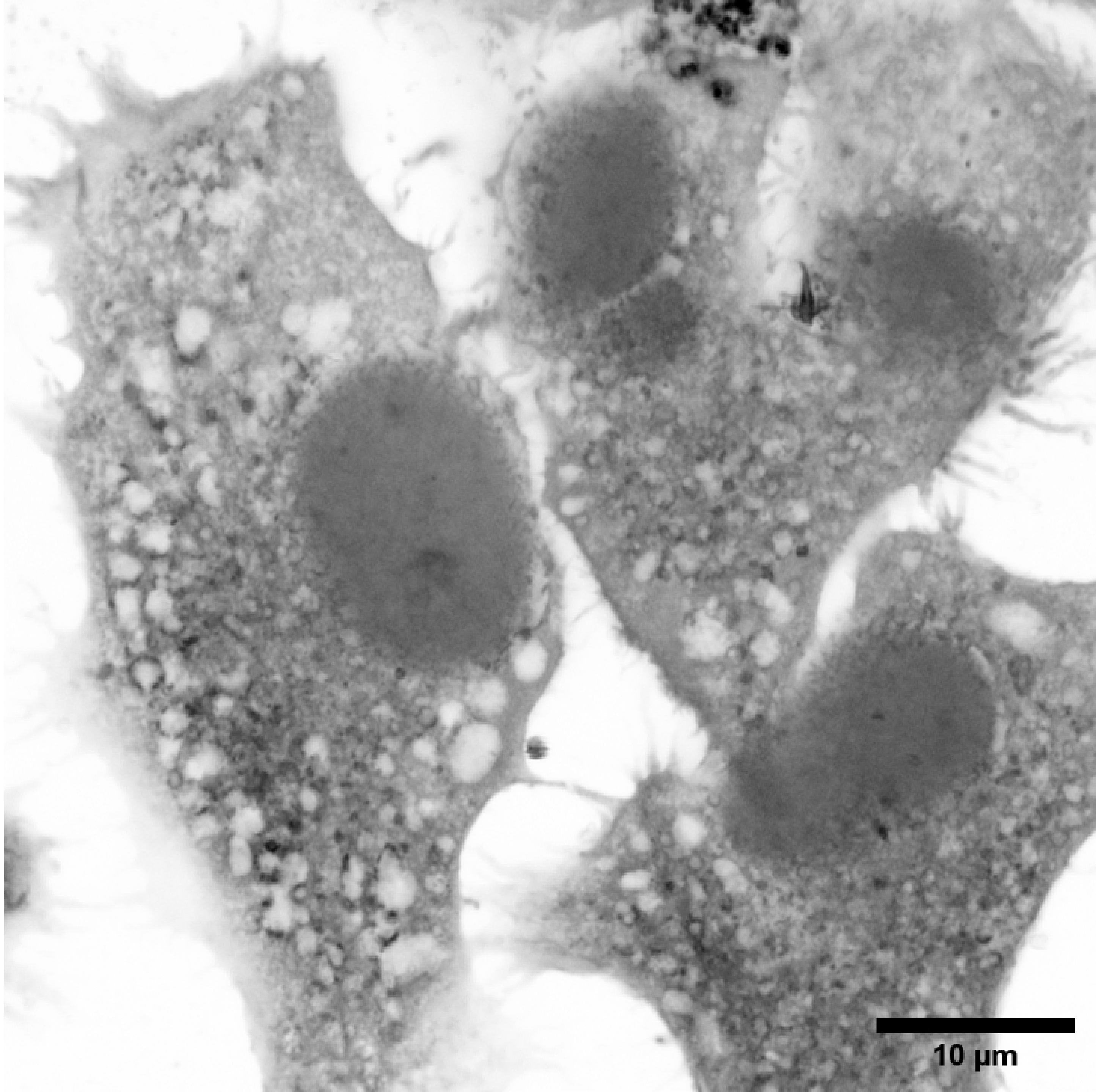

NHS AZ532

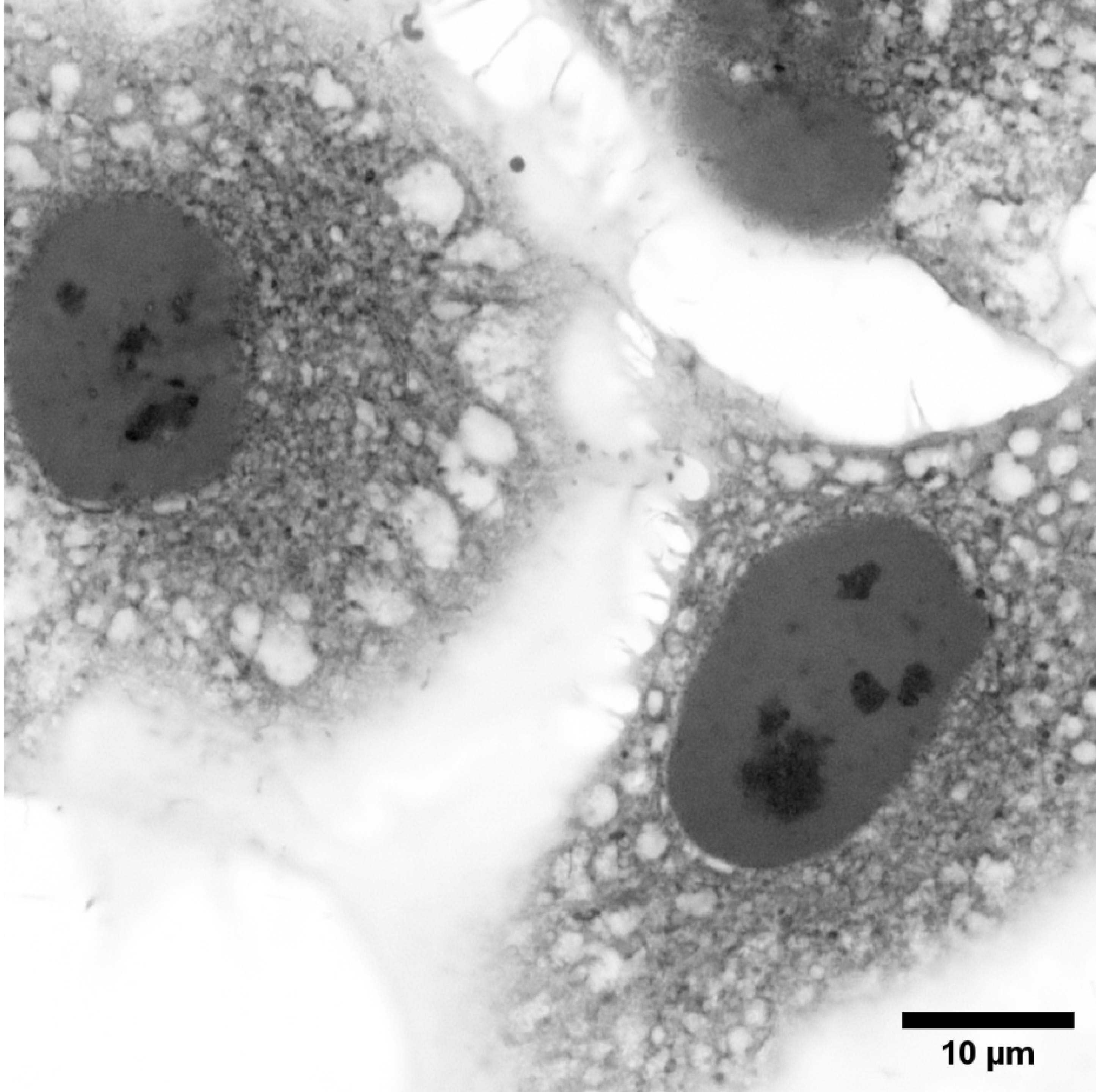

NHS AZ647

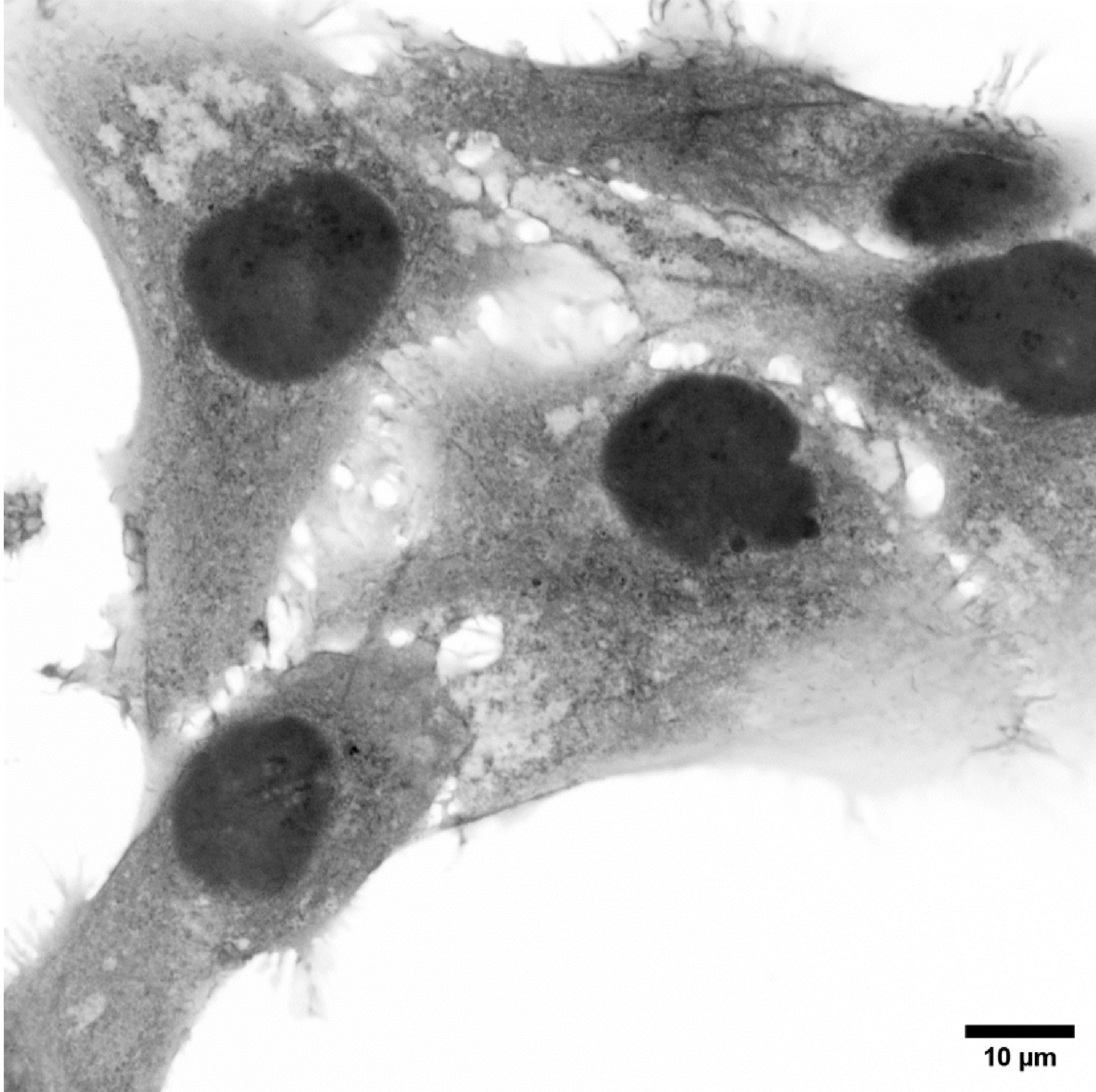

NHS MB543

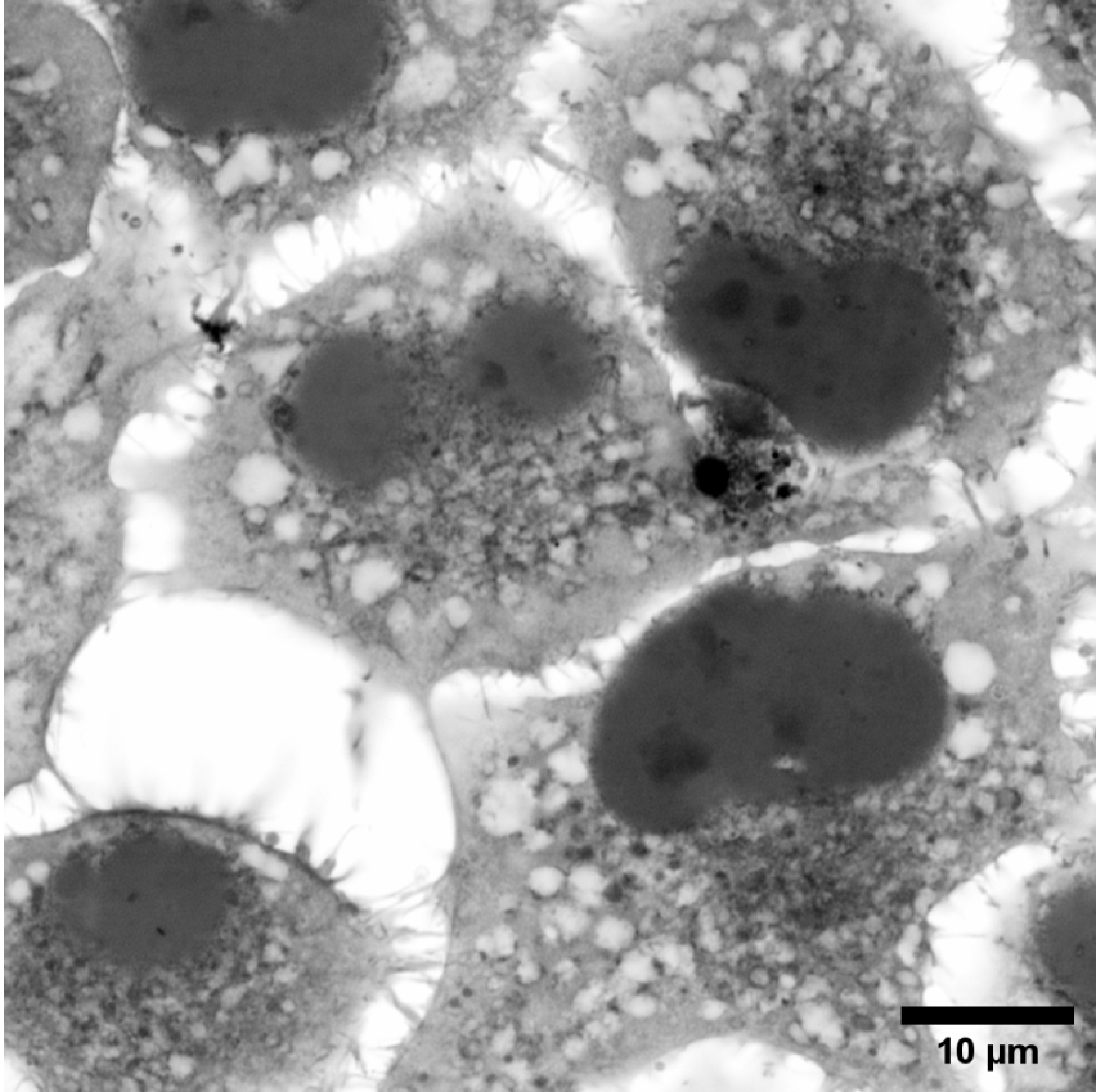

NHS MB660R

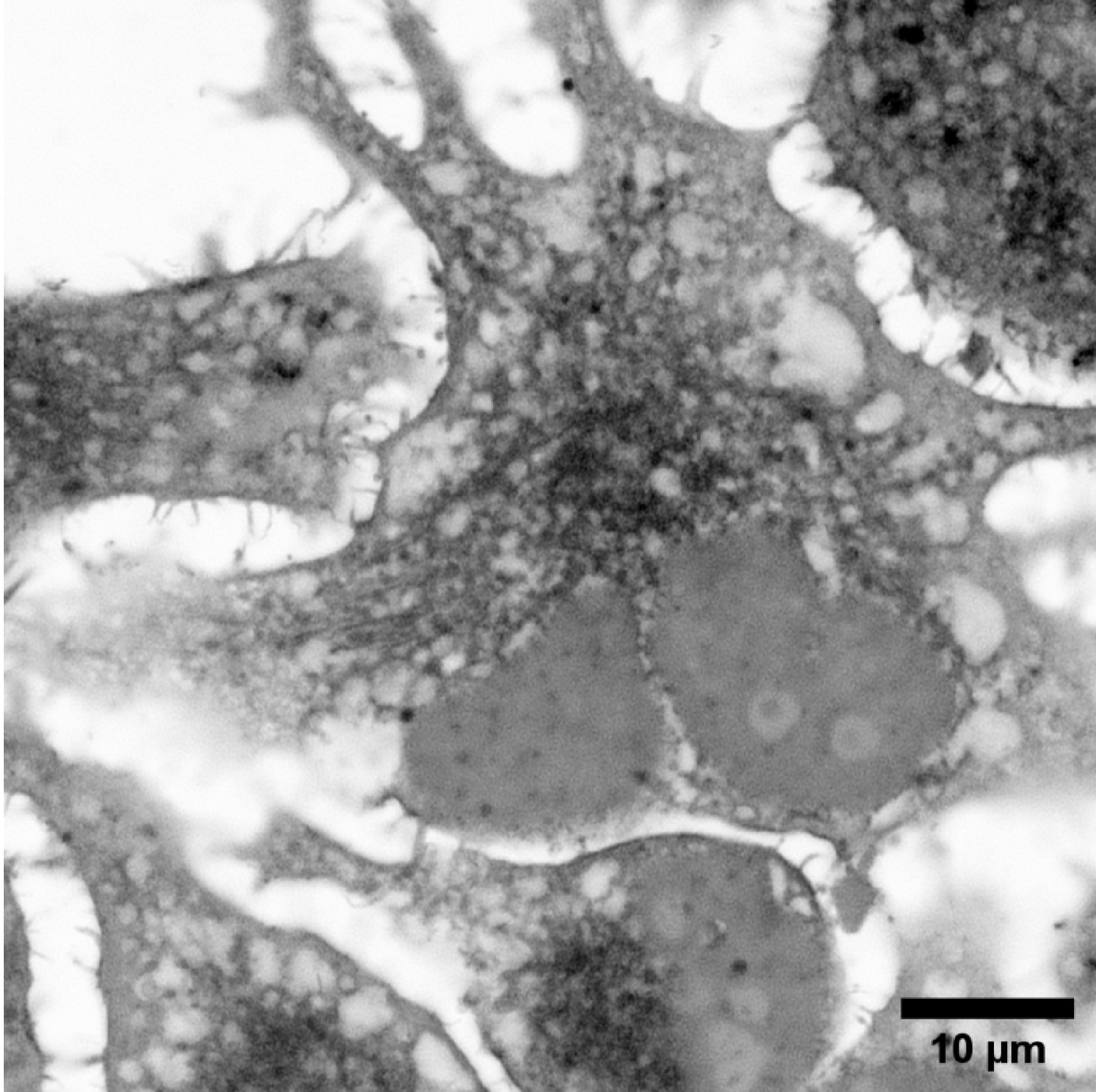

NHS BODIPY493/503

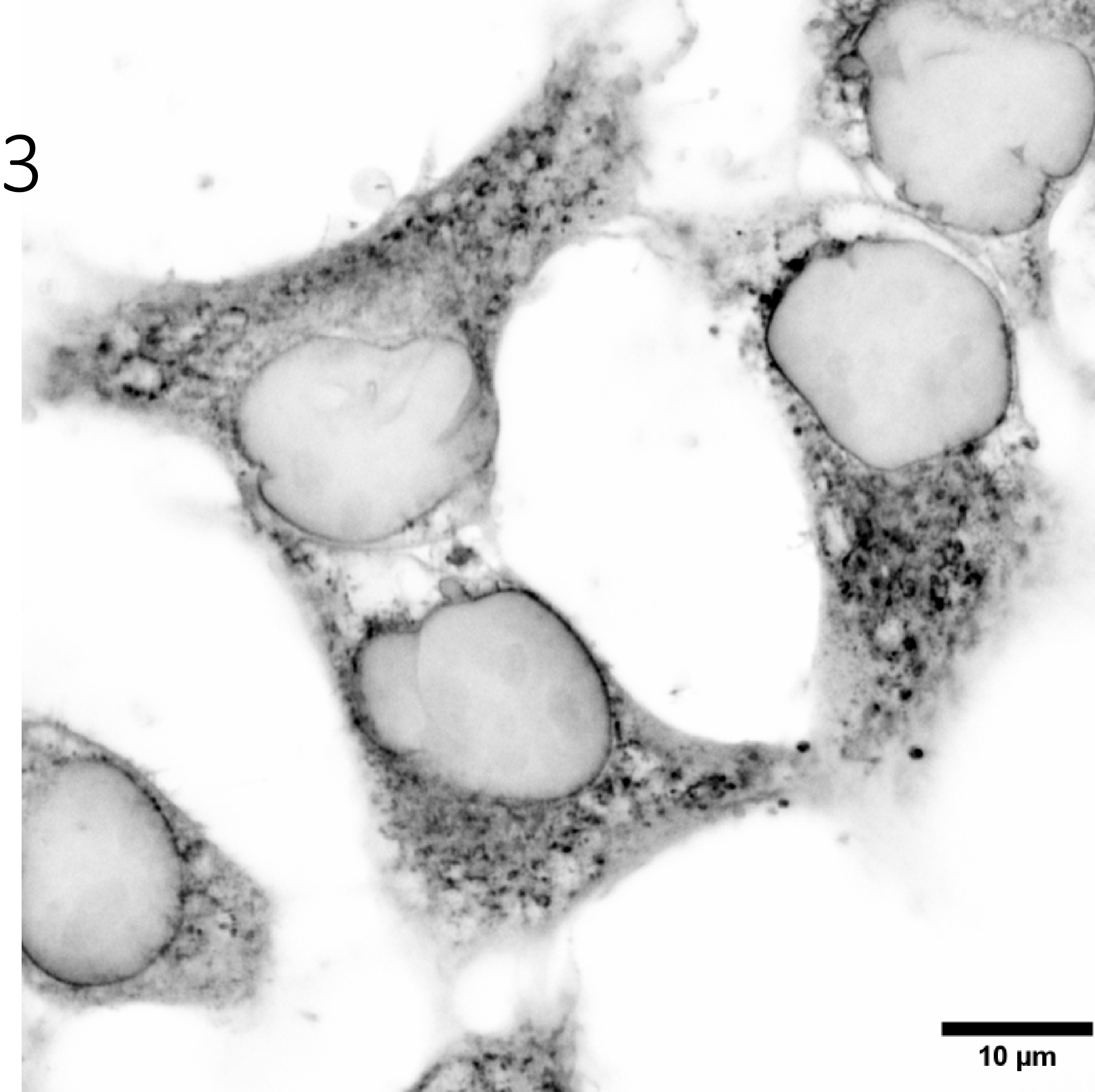

NHS BODIPY581/591

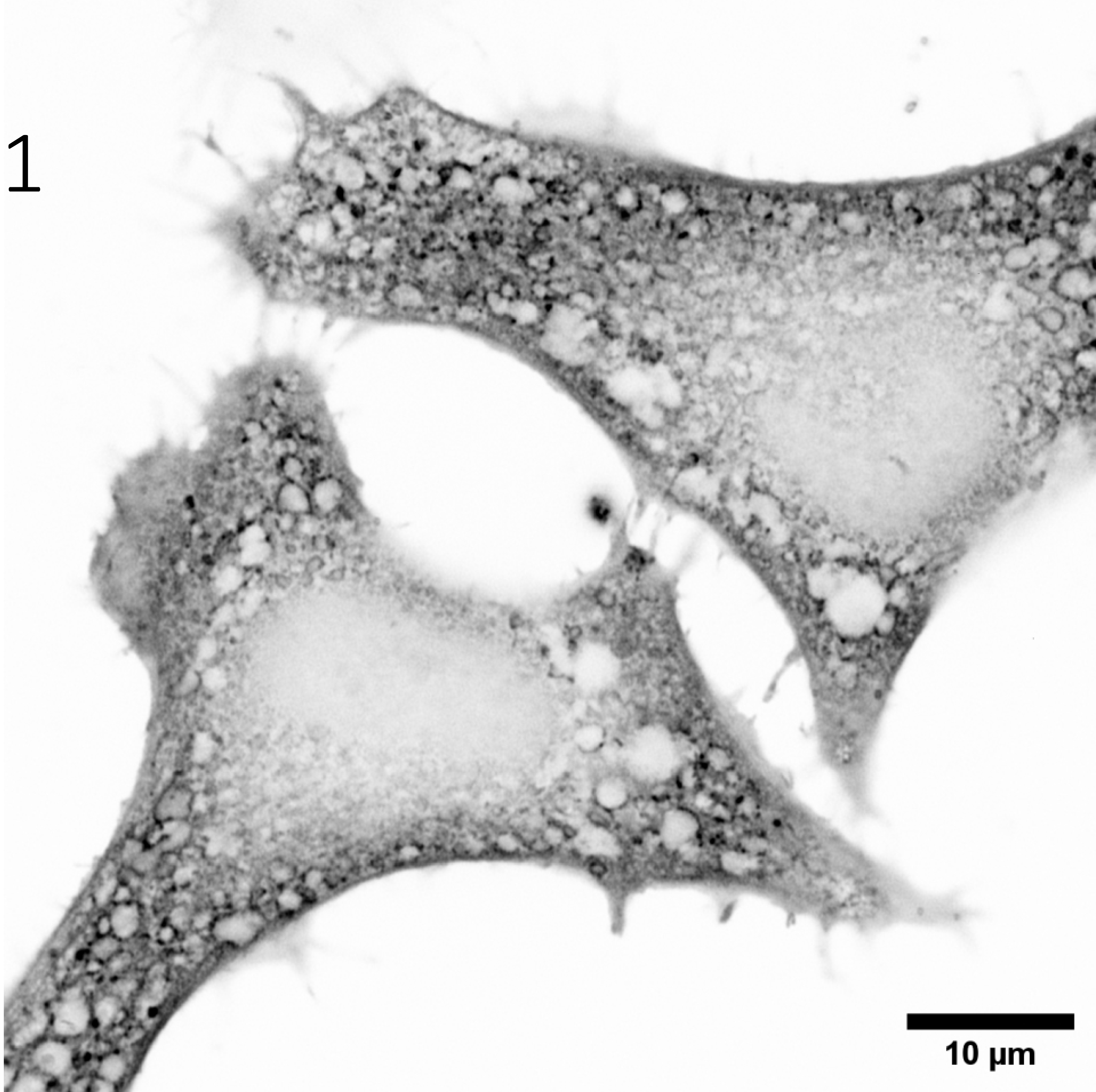

NHS BODIPY630/650-X

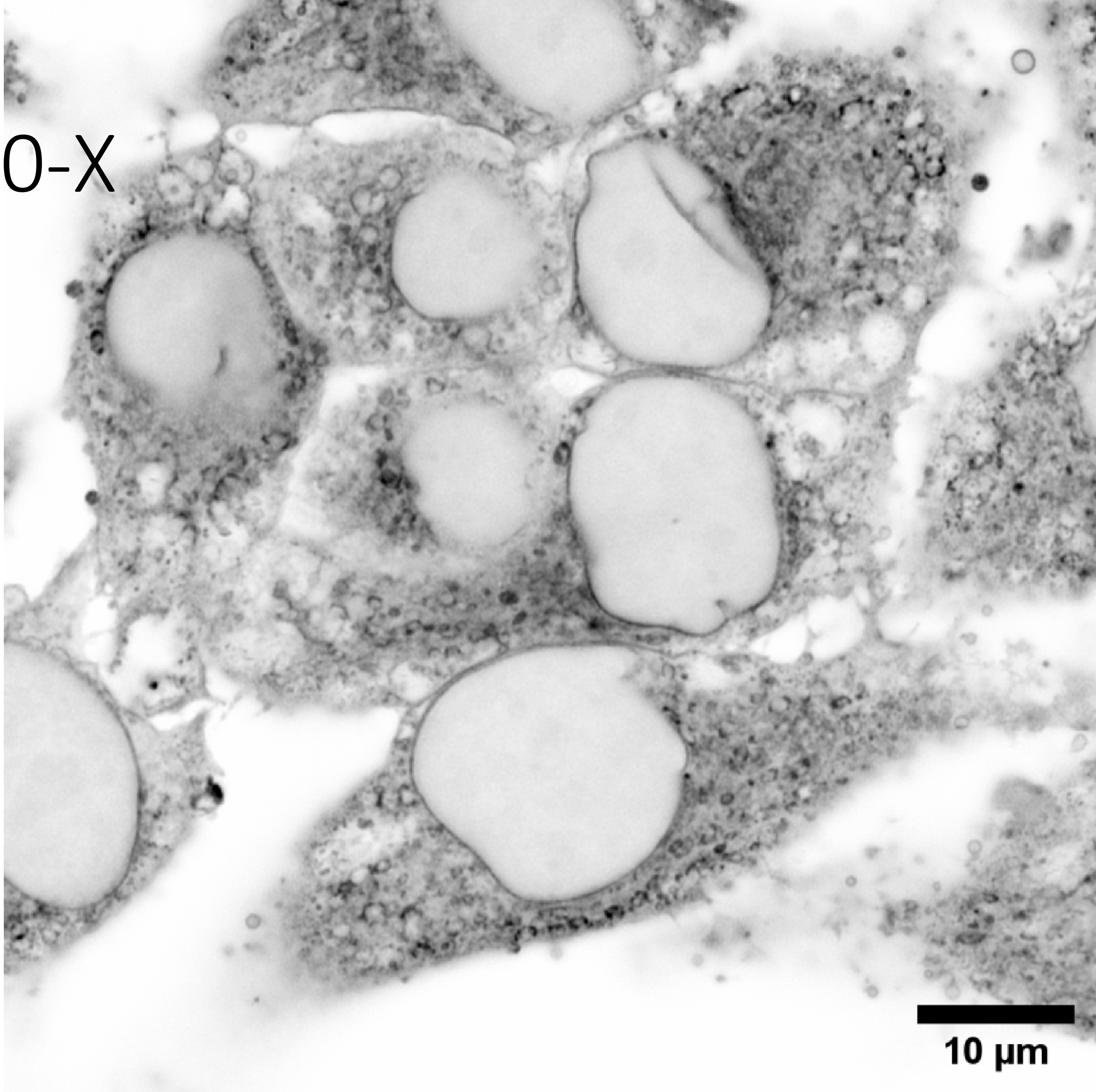

NHS ATTO425

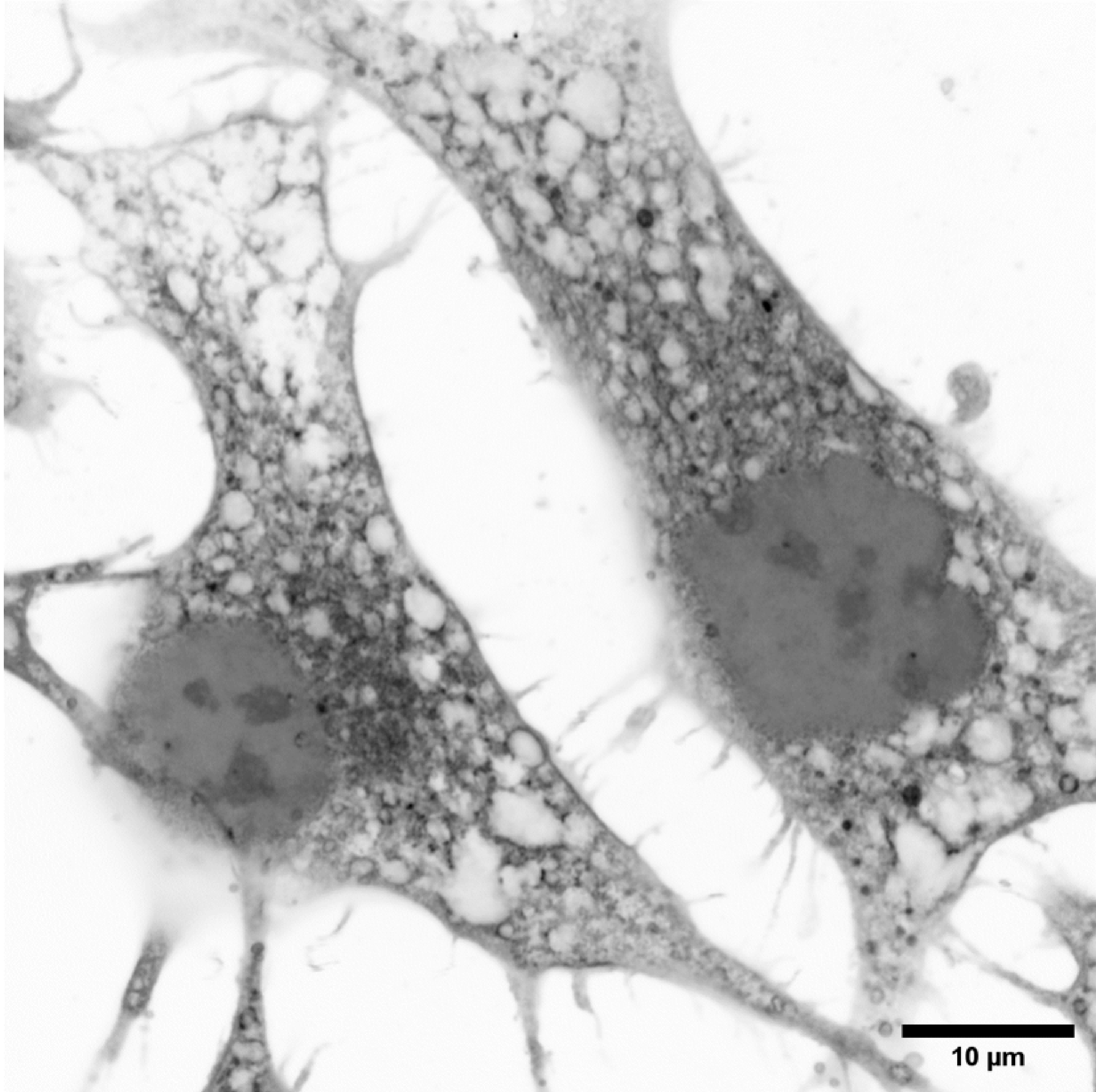

NHS ATTO594

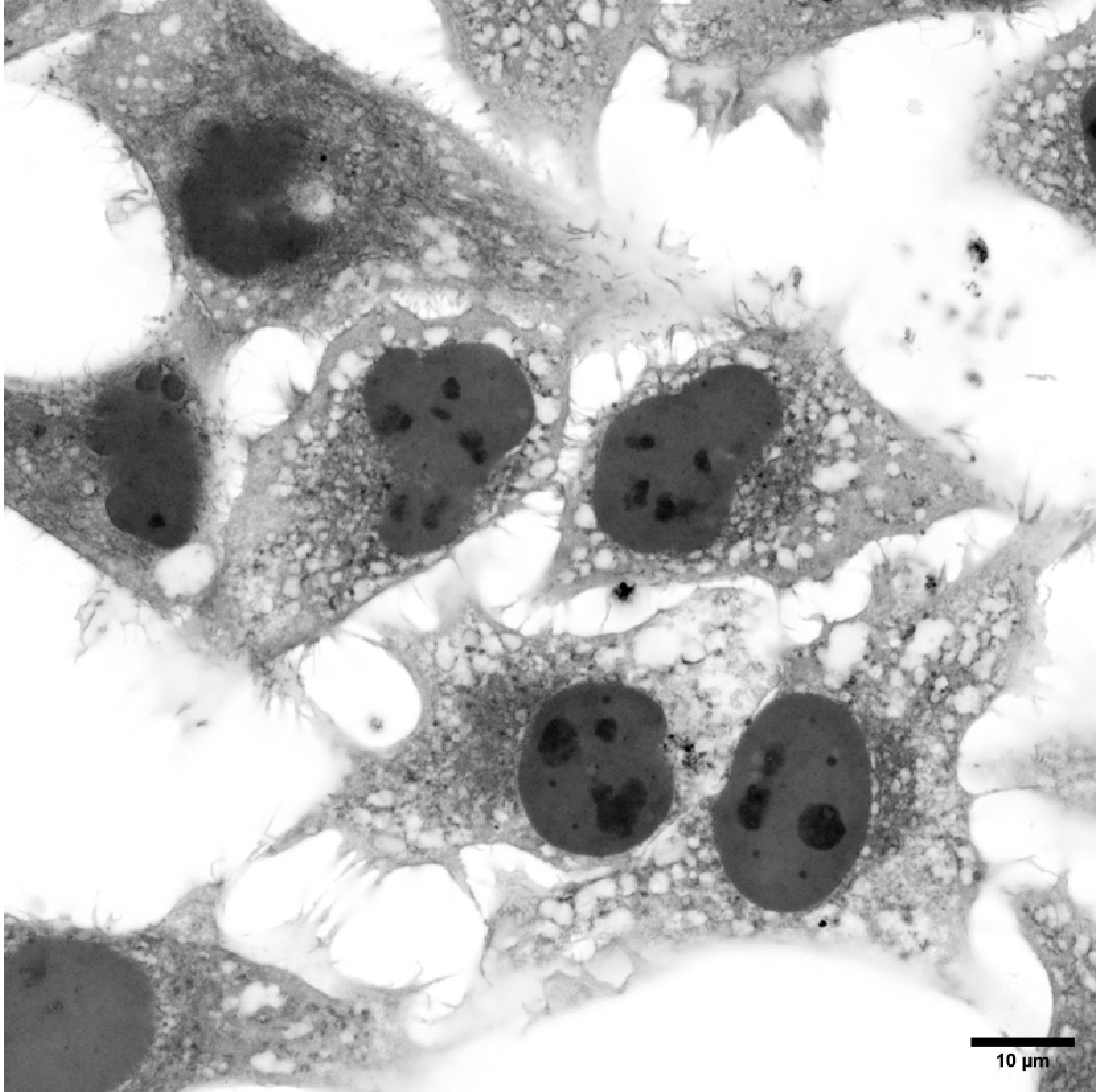

NHS ATTO647N

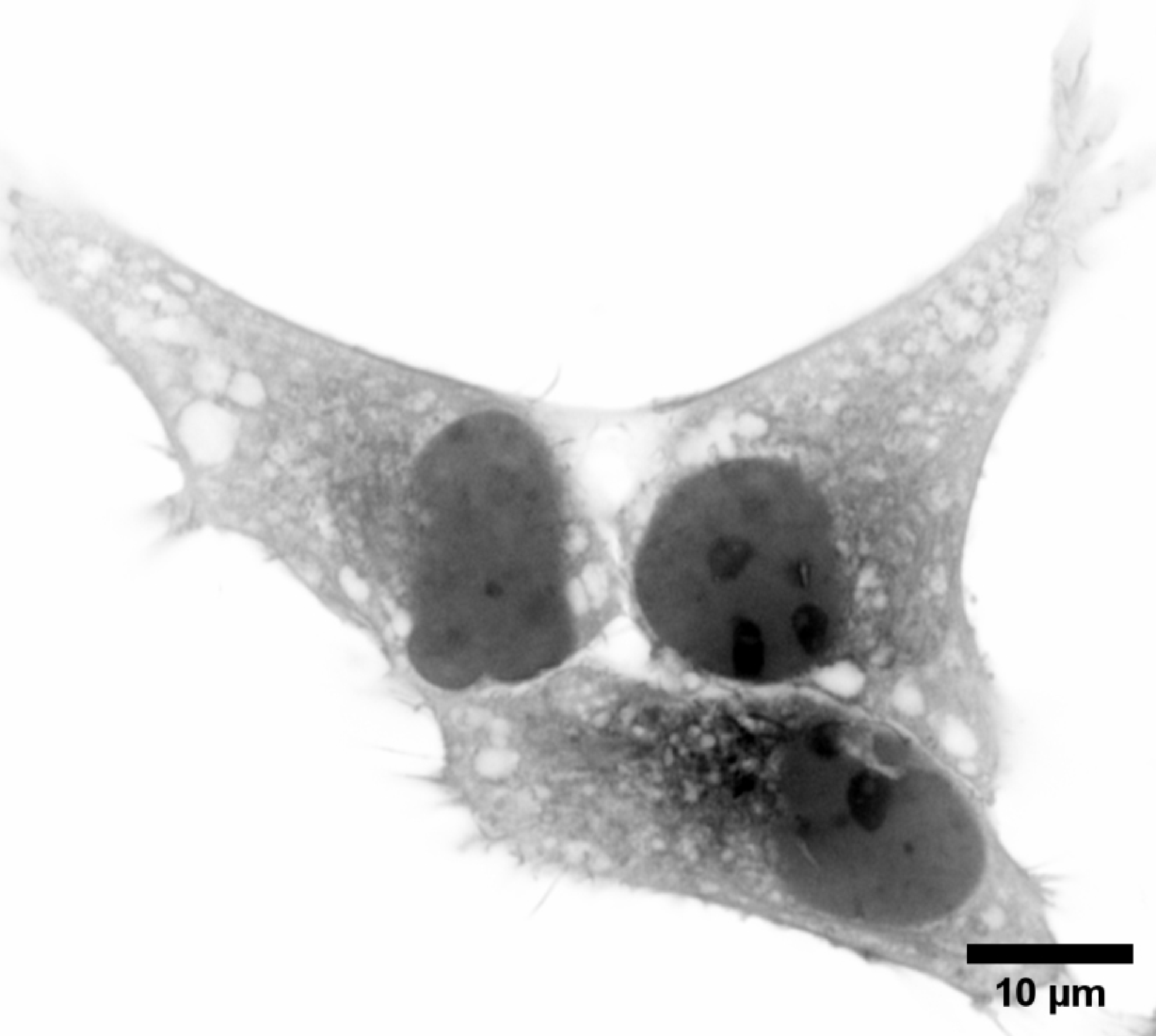

Supplement: NR-015-D3NR01129A-s001 [file NR-015-D3NR01129A-s001.pdf]
