# Supplementary material for: Differential labelling of human sub-cellular compartments with fluorescent dye esters and expansion microscopy
Source: Nanoscale. 2023 Nov 9;15(45):18489–99. doi: 10.1039/d3nr01129a (PMC10667587; doi:10.1039/d3nr01129a)
Supplement: NR-015-D3NR01129A-s002 [file NR-015-D3NR01129A-s002.pdf]

Electronic Supplementary Material (ESI) for Nanoscale.
This journal is © The Royal Society of Chemistry 2023

SUPPLEMENTARY INFORMATION

Differential labelling of human sub-cellular compartments with fluorescent dye esters and expansion microscopy

Authors:

Thomas M. D. Sheard[1,*], Tayla B. Shakespeare[1], Rajpinder S. Seehra[1], Michael E Spencer[1], Kin M. Suen[2] and Izzy Jayasinghe[1,3,*]

Affiliations:

1. School of Biosciences, Faculty of Science, University of Sheffield, Sheffield S10 2TN, UK.

2. School of Molecular and Cellular Biology, University of Leeds, LS2 9JT, UK.

3. School of Biomedical Sciences, University of New South Wales, Kensington 2052, NSW, Australia.

* Correspondence to either i.jayasinghe@sheffield.ac.uk or t.sheard@sheffield.ac.uk

Supplementary figures

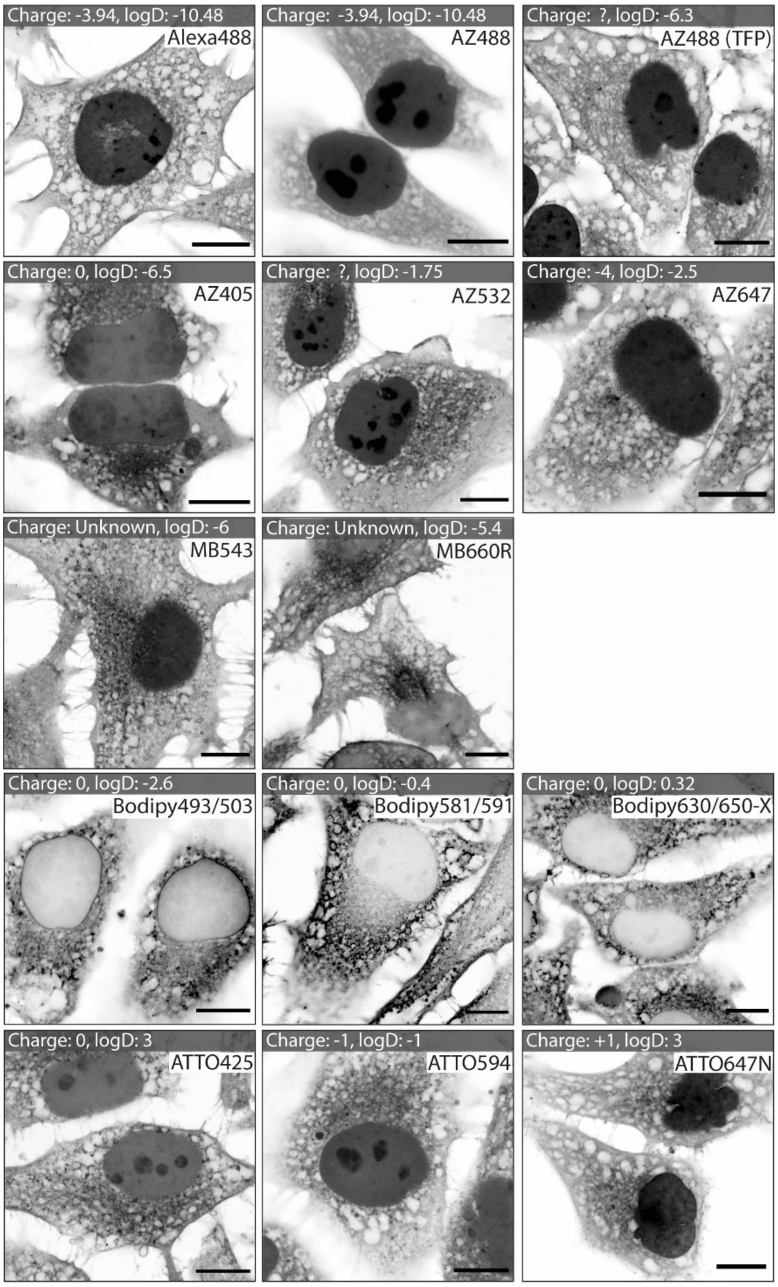

Supplementary figure 1. Catalogue of unexpanded dye ester images. (A) Chart displaying the ester dyes, distributed based on their excitation wavelength and hydrophobicity (determined from logD values). **(B)** A gallery of unexpanded images of each of the esters in HeLa cells. Between different dye families there are substantial differences, and within a single dye family there are similarities in patterns. Scale bars: 10 µm.

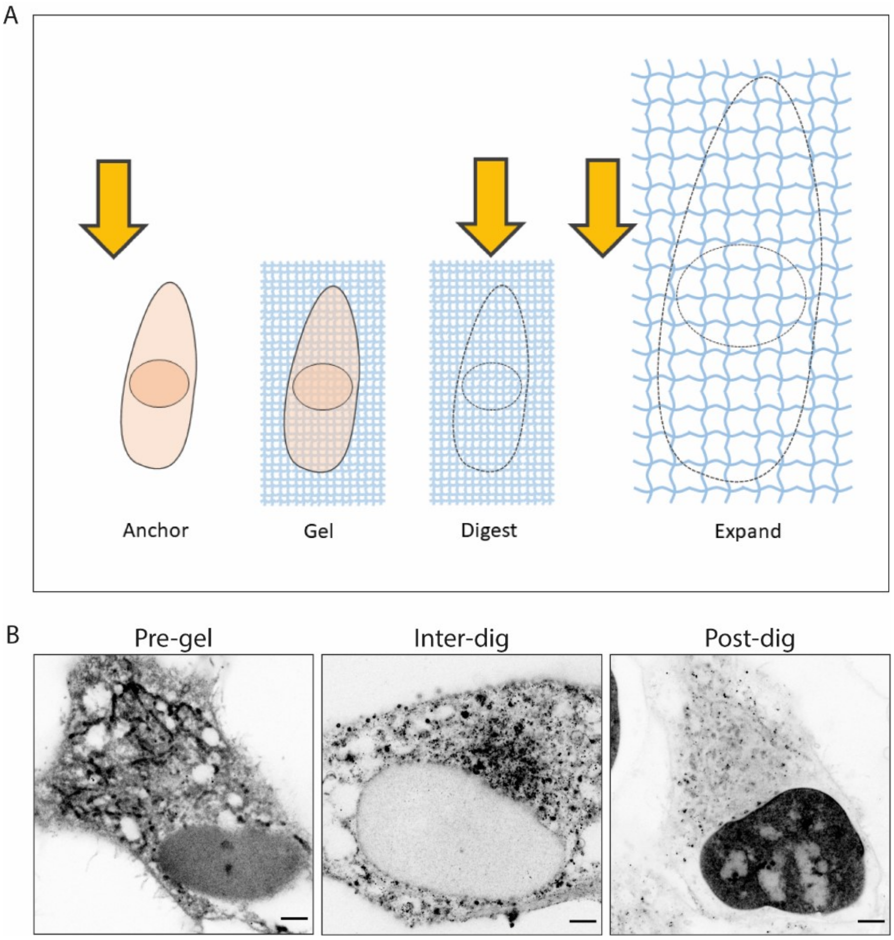

Supplementary figure 2. (A) Schematic showing the generalised ExM pipeline. Ester labelling can be performed at different timepoints (highlighted by yellow arrows) either pre-gelation, inter-digestion (4 hours of digestion, ester labelling, another 4 hours digestion), or post-digestion. **(B)** 4x EExM images of NHS ATTO647N in HeLa cells applied either pre-gelation, inter-digestion (4 hours of digestion, ester labelling, another 4 hours of digestion), or post-digestion, demonstrate the variety of intracellular structures which can be targeted. Scale bars (expansion factor rescaled): 2.5 µm.

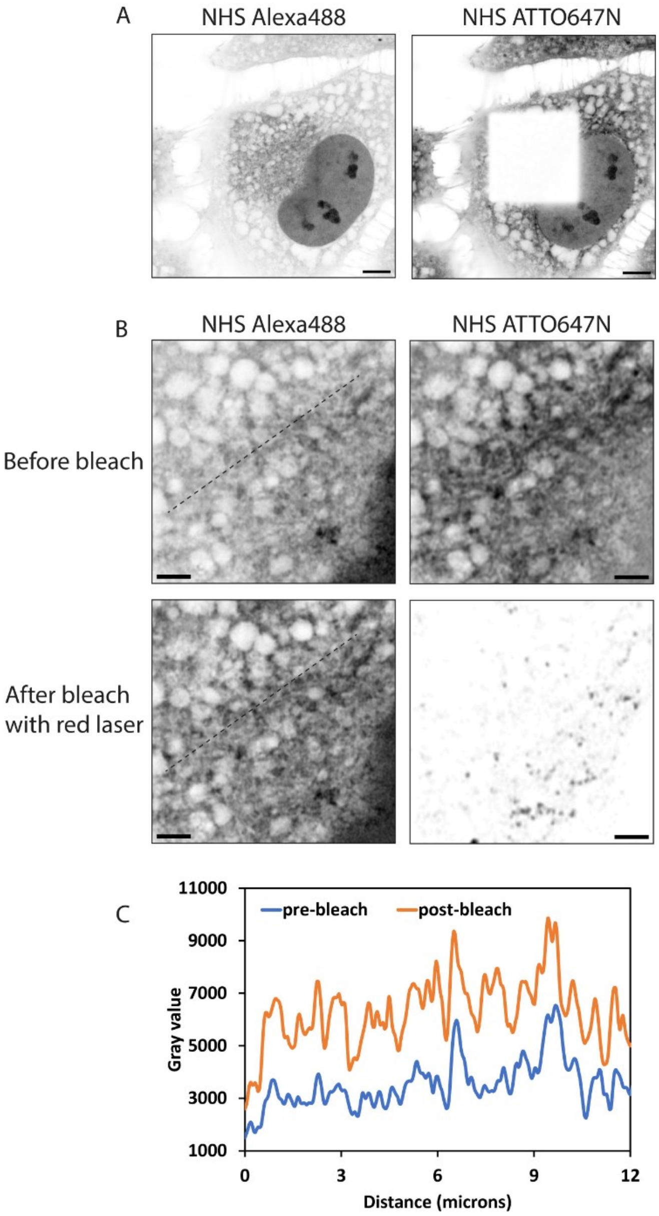

Supplementary figure 3. Observation of fluorescence resonance energy transfer (FRET) interaction between multiple esters in unexpanded samples. A HeLa cell sample was labelled with NHS Alexa488 and NHS ATTO647N. **(A)** After bleaching a square in the NHS ATTO647N channel using a 639 nm laser, by zooming out it is possible to see that the intensity of NHS Alexa488 in the same region is increased in intensity **(B)**. Images of the region before and after bleaching with a 639 nm laser. **(C)** Line profile demonstrates the increase in pixel intensity post-bleach. Scale bars: (A) 5 µm; (B) 2 µm.

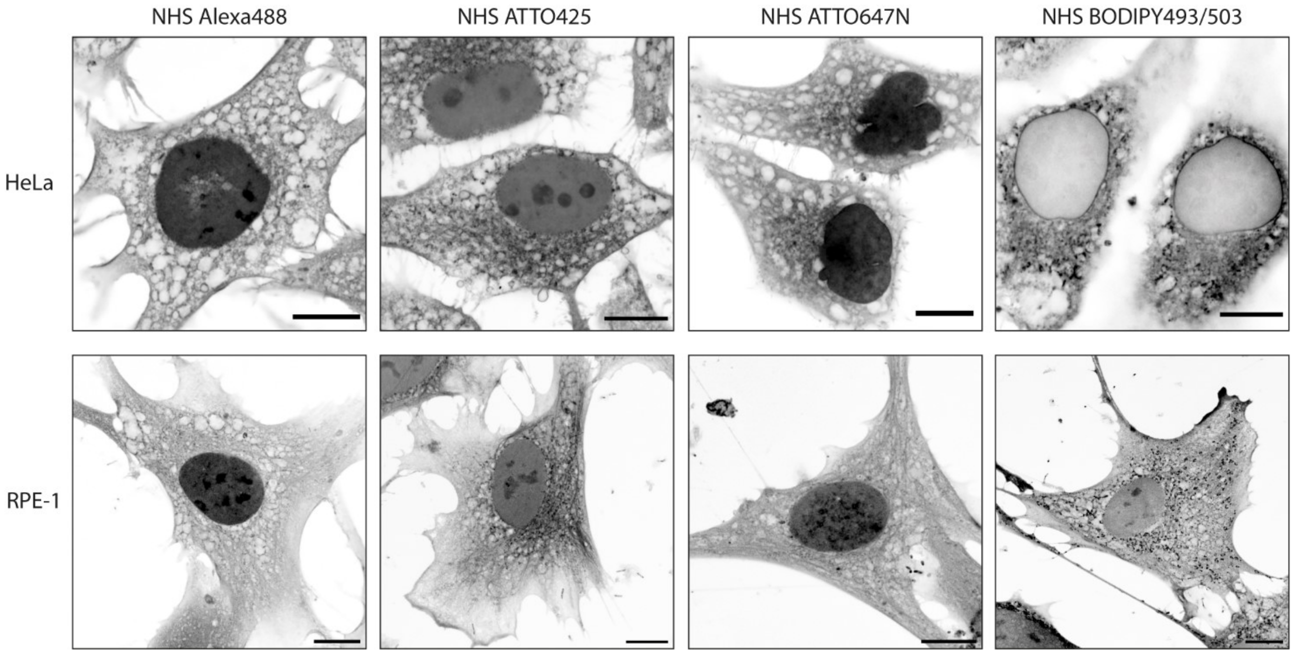

Supplementary figure 4. Unexpanded images of four NHS esters in HeLa and RPE1 cells. Scale bars: 10 µm.

Supplementary dataset

Additional images of unexpanded HeLa cells labelled with each ester are provided in the PowerPoint file, to demonstrate the consistency of labelling patterns across a wider selection of cells. The images were acquired with the LSM 880 Airyscan microscope (Carl Zeiss, Jena), using a 40x oil immersion 1.3 NA objective.

Tables

Table 1. Ester properties.

Details of each ester-dye conjugate are provided, specifically the light maximum excitation and emission wavelengths, and the chemical properties of hydrophobicity (inferred from the logD value, D being the ratio of the solute between a nonpolar and a polar solvent) and the charge. The more positive value of logD, the more hydrophobic the molecule. Information was obtained from the following sources: [a](Zanetti-Domingues et al., 2013), [b](Hughes et al., 2014), [c](Zhang et al., 2017), [d](URL: https://www.spectra.arizona.edu/supplemental/ATTO_Dye_Properties_01.pdf, accessed 15/02/2023), *(calculated using Chemaxon logD predictor).

Dye	Exc. Max (nm)	Em. Max (nm)	Hydrophobicity (logD)	Overall Charge	Source
NHS Alexa488	494	517	(-10.48) [a]	(-3.94)[a]	Thermo Fisher Scientific
NHS AZ488	490	525	(-9.4)*	(-3.94)[a]	Fluoroprobes
TFP AZ488	495	515	(-6.3)*	Unknown	Fluoroprobes
NHS AZ405	401	421	(-6.5)*	Unknown	Fluoroprobes
NHS AZ532	532	554	(-3.26) [b], (-1.75)*	Unknown	Fluoroprobes
NHS AZ647	650	665	(-6.72) [b], (-2.5)*	-(4)[c]	Fluoroprobes
NHS BODIPY493/503	493	503	(-2.6)*	0	Thermo Fisher Scientific
NHS BODIPY581/591	581	591	(-0.4)*	0	Thermo Fisher Scientific
NHS BODIPY630/ 650-X	625	640	(0.32)*	0	Thermo Fisher Scientific
NHS ATTO425	439	485	(3)*	(0)[d]	Sigma-Aldrich
NHS ATTO594	597	625	(-1)*	(-1)[d]	Sigma-Aldrich
NHS ATTO647N	645	669	(1.96) [a],(3)*	(1)[d], (0.61)[a]	Sigma-Aldrich
NHS MB543	543	566	(-6)*	1 more negative sulfo group vs equivalent Alexa dye	Fluoroprobes
NHS MB660R	673	694	(-5.4)*	1 more negative sulfo group vs equivalent Alexa dye	Fluoroprobes

Table 2. Key resources.

	SOURCE	IDENTIFIER
**Experimental models: Cell lines**		
Human: HeLa cell	Sigma	Cat# 93021013
Human: hTERT retinal pigment epithelial cell	ATCC	Cat# CRL-4000
**Chemicals**		
Dulbecco's Modified Eagle Medium	Thermo Fisher Scientific	Cat# 11584496
Dulbecco's modified Eagle's medium nutrient mixture F12 Ham	Sigma-Aldrich	Cat# D8062
Non-essential amino acids 100x	Thermo Fisher Scientific	Cat# 11140050
Foetal bovine serum	Labtech	Cat# SKU: FB-1001/100-500
Penicillin-streptomycin	Thermo Fisher Scientific	Cat# 11548876
Poly-D-lysine	Cultrex	Cat# 3439-100-01
Paraformaldehyde	Sigma-Aldrich	Cat# P6148-500G
Bovine serum albumin	Thermo Fisher Scientific	Cat# 30063-572
Sodium azide	Sigma-Aldrich	Cat# S8032-25G
DMSO	Biotium	Cat# 90082-BT
Triton X-100	Sigma-Aldrich	Cat# T9284-100ML
Normal goat serum	Thermo Fisher Scientific	Cat# 10000C
Acryloyl-X	Thermo Fisher Scientific	Cat# A20770
Acrylamide	Sigma-Aldrich	Cat# A9099-25G
N,N'-Methylenebisacrylamide	Sigma-Aldrich	Cat# M7279-25G
Ammonium persulfate	Sigma-Aldrich	Cat# A3678-25G
N,N,N',N'-Tetramethylethylenediamine	Sigma-Aldrich	Cat# T7024-25ML
Proteinase K	New England Biolabs	Cat# P8107S
Ethylenediaminetetraacetic acid	Sigma-Aldrich	Cat# EDS-100G

Guanidine HCl	Sigma-Aldrich	Cat# G3272-25G
Tris pH 8.0	Invitrogen	Cat# P8920-100ML
Poly-L-lysine	Sigma-Aldrich	Cat# 10259194
**Dye-esters**		
NHS Alexa488	Thermo Fisher Scientific	Cat# a20000
NHS AZ488	Fluoroprobes	Cat# 1013-1
TFP AZ488	Fluoroprobes	Cat# 1026
NHS AZ405	Fluoroprobes	Cat# 1061-1
NHS AZ532	Fluoroprobes	Cat# 1041-1
NHS AZ647	Fluoroprobes	Cat# 1121-1
NHS BODIPY493/503	Thermo Fisher Scientific	Cat# D2191
NHS BODIPY581/591	Thermo Fisher Scientific	Cat# D2228
NHS BODIPY630/650-X	Thermo Fisher Scientific	Cat# D10000
NHS ATTO425	Sigma-Aldrich	Cat# 16805
NHS ATTO594	Sigma-Aldrich	Cat# 8741
NHS ATTO647N	Sigma-Aldrich	Cat# 18373
NHS MB543	Fluoroprobes	Cat# 1661-1
NHS MB660R	Fluoroprobes	Cat# 1661-1
**Antibodies**		
Rabbit polyclonal KDEL	Thermo Fisher Scientific	Cat# PA1-013
Rabbit monoclonal GM130	Abcam	Cat# ab52649
Mouse monoclonal ATP5A1	Thermo Fisher Scientific	Cat# 43-9800
Alexa Fluor 488 goat anti-mouse IgG	Thermo Fisher Scientific	Cat# A11001
Alexa Fluor 488 goat anti-rabbit IgG	Thermo Fisher Scientific	Cat# A11008
Alexa Fluor 594 goat anti-mouse IgG	Thermo Fisher Scientific	Cat# A11005
Alexa Fluor 594 goat anti-rabbit IgG	Thermo Fisher Scientific	Cat# A11012

Atto 647N goat anti-rabbit IgG	Sigma-Aldrich	Cat# 40839-1ML-F
Atto 647N goat anti-mouse IgG	Sigma-Aldrich	Cat# 50185-1ML-F
**Items**		
Glass coverslips, #1.5, 22x22 mm	Menzel-Glaser	Cat# 631-0851
Chamberslides	Razorlab	Custom-made
**Software and algorithms**		
Fiji	ImageJ	https://imagej.net/software/fiji/
Chemaxon calculator plugin	Chemaxon	https://disco.chemaxon.com/calculators/demo/plugins/logd/
Excel	Microsoft	
