# Supplementary figures and images for: Differential labelling of human sub-cellular compartments with fluorescent dye esters and expansion microscopy
Source: Nanoscale. 2023 Nov 9;15(45):18489–99. doi: 10.1039/d3nr01129a (PMC10667587; doi:10.1039/d3nr01129a)

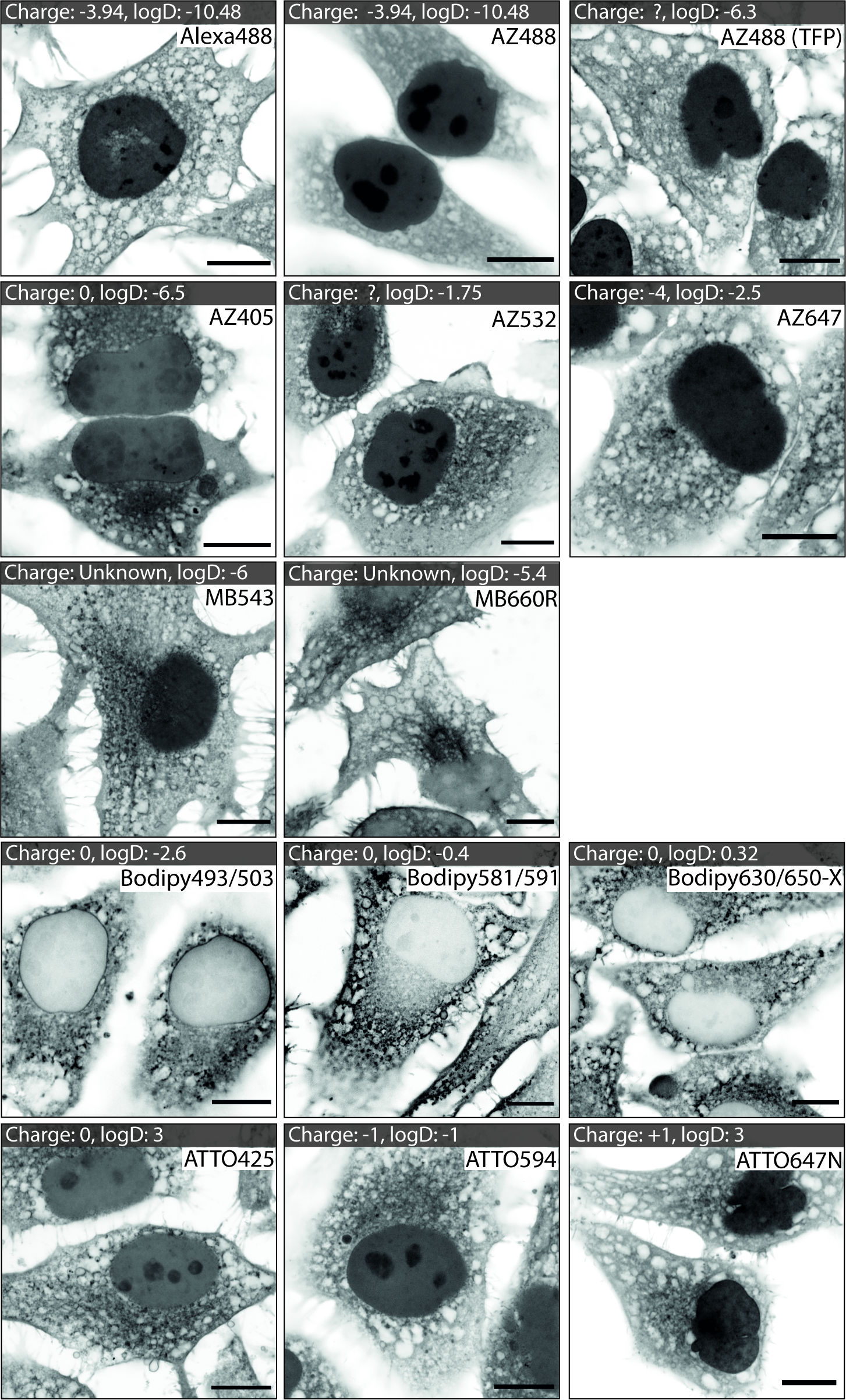

Supplement: NR-015-D3NR01129A-s003 [file NR-015-D3NR01129A-s003.zip › Suppl 1 Ester catalogue unexpanded.tif]

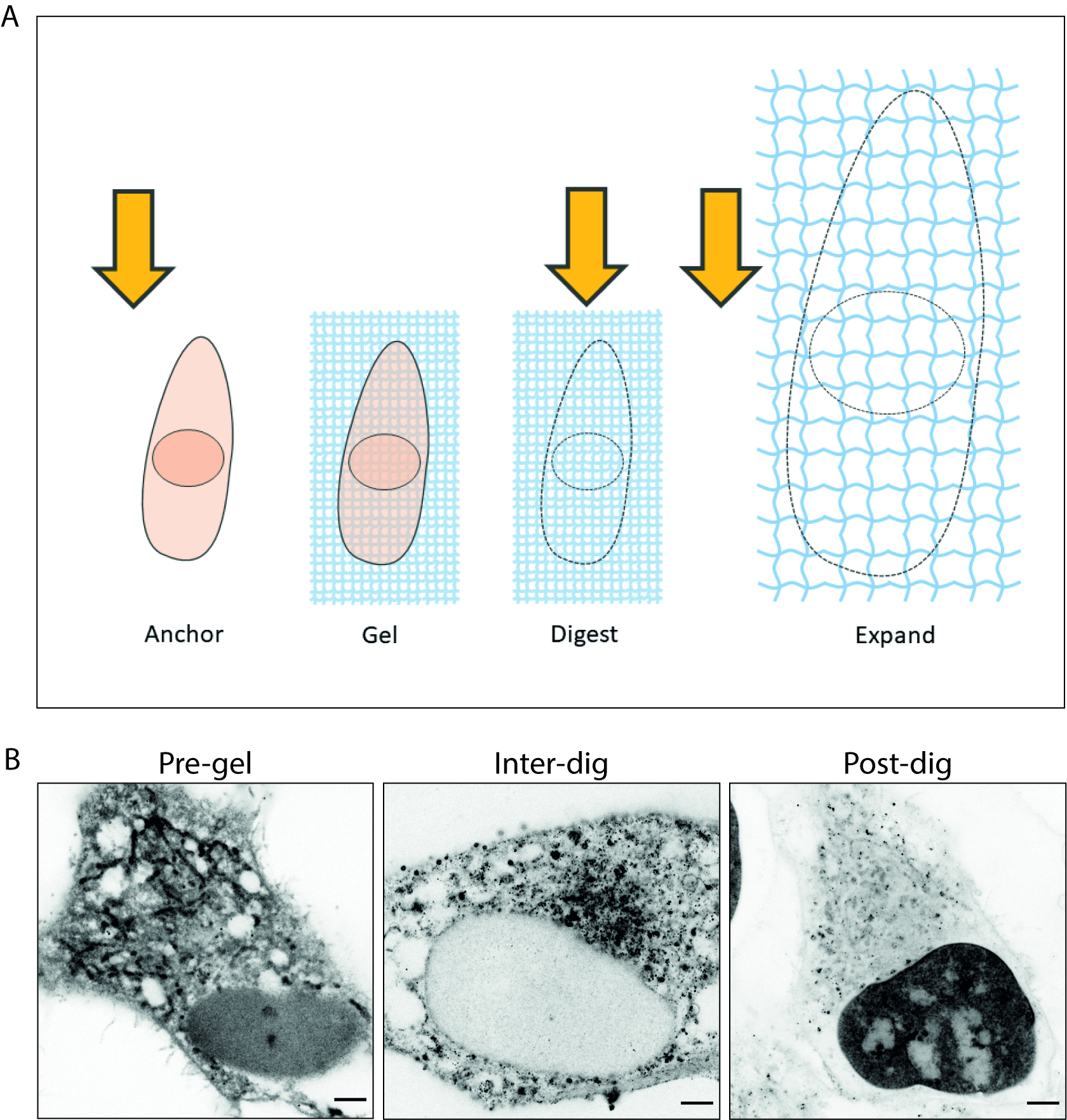

Supplement: NR-015-D3NR01129A-s003 [file NR-015-D3NR01129A-s003.zip › Suppl 2 Figure Timing.tif]

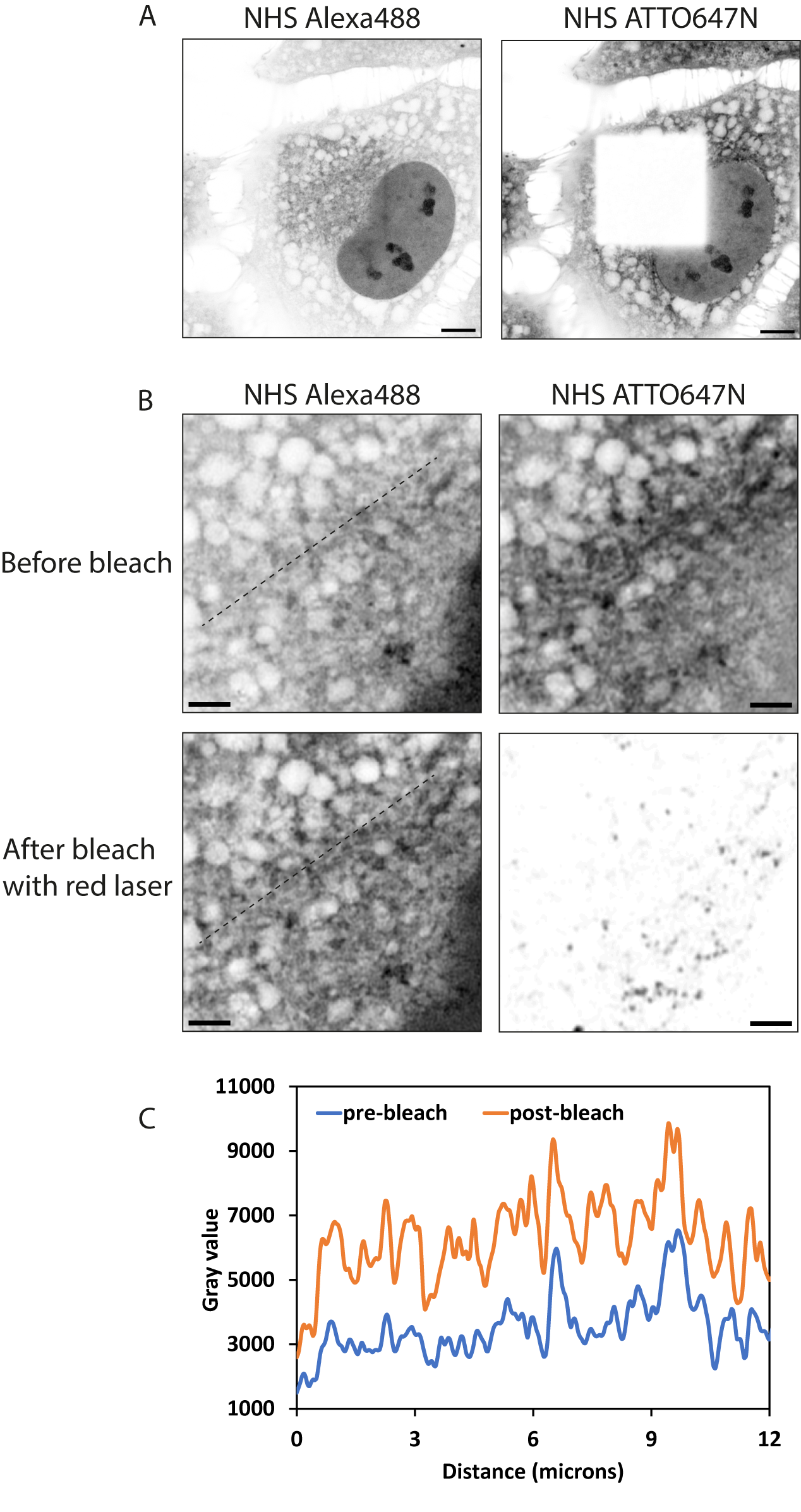

Supplement: NR-015-D3NR01129A-s003 [file NR-015-D3NR01129A-s003.zip › Suppl 3 FRET.tif]

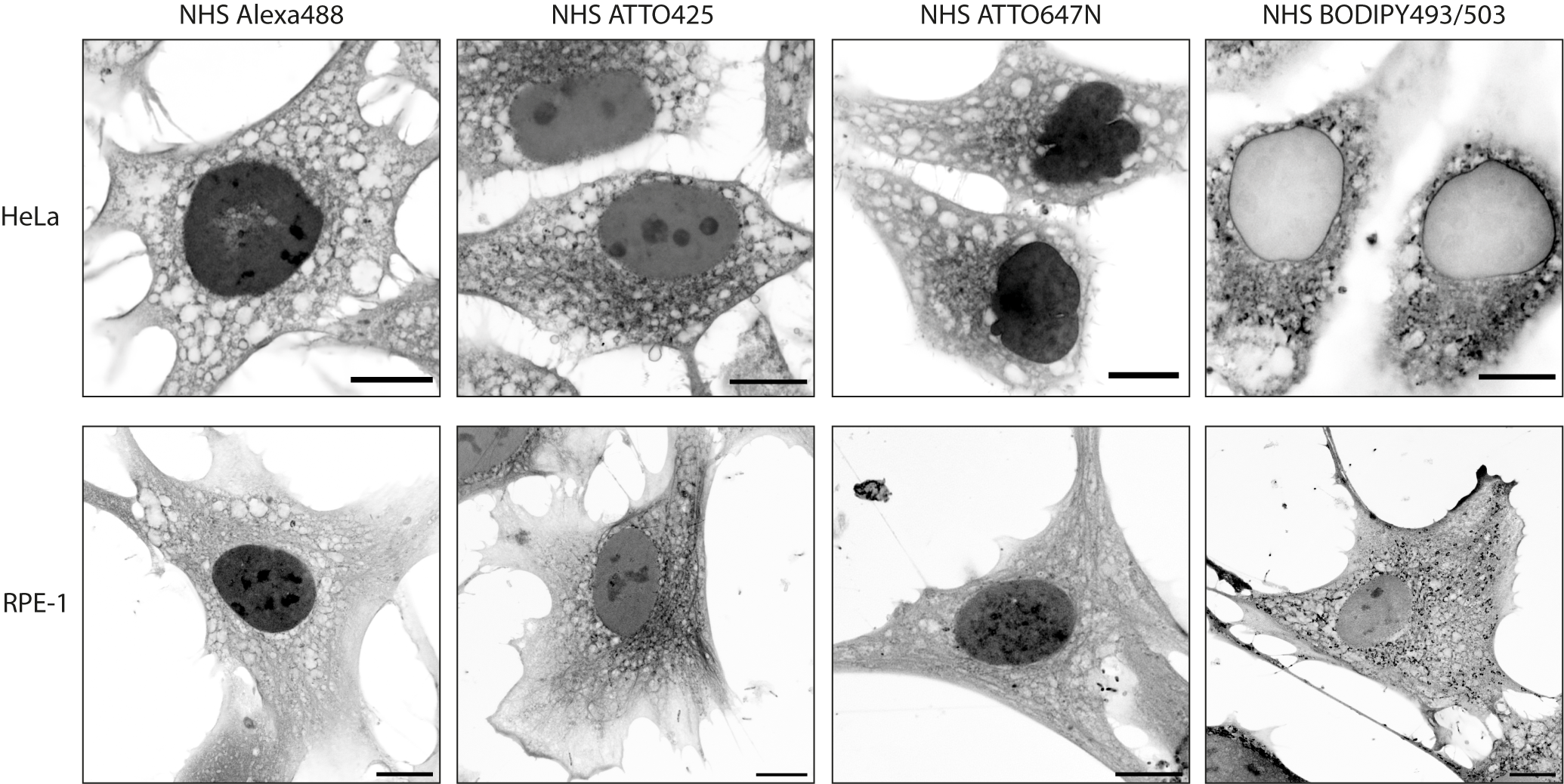

Supplement: NR-015-D3NR01129A-s003 [file NR-015-D3NR01129A-s003.zip › Suppl 4 RPE1.tif]
